# Transcriptional inaccuracy threshold attenuates differences in RNA-dependent DNA synthesis fidelity between retroviral reverse transcriptases

**DOI:** 10.1038/s41598-017-18974-8

**Published:** 2018-01-12

**Authors:** Alba Sebastián-Martín, Verónica Barrioluengo, Luis Menéndez-Arias

**Affiliations:** 1 Centro de Biología Molecular “Severo Ochoa” (Consejo Superior de Investigaciones Científicas & Universidad Autónoma de Madrid), c/Nicolás Cabrera, 1, Campus de Cantoblanco, 28049 Madrid, Spain; 2 Present Address: DiaSorin Iberia S.A., Avenida de la Vega 1, 28108 Alcobendas (Madrid), Spain

## Abstract

In M13mp2 *lacZα* forward mutation assays measuring intrinsic fidelity of DNA-dependent DNA synthesis, wild-type human immunodeficiency virus type 1 (HIV-1) RTs of group M/subtype B previously showed >10-fold higher error rates than murine leukaemia virus (MLV) and avian myeloblastosis virus (AMV) RTs. An adapted version of the assay was used to obtain error rates of RNA-dependent DNA synthesis for several RTs, including wild-type HIV-1_BH10_, HIV-1_ESP49_, AMV and MLV RTs, and the high-fidelity mutants of HIV-1_ESP49_ RT K65R and K65R/V75I. Our results showed that there were less than two-fold differences in fidelity between the studied RTs with error rates ranging within 2.5 × 10^−5^ and 3.5 × 10^−5^. These results were consistent with the existence of a transcriptional inaccuracy threshold, generated by the RNA polymerase while synthesizing the RNA template used in the assay. A modest but consistent reduction of the inaccuracy threshold was achieved by lowering the pH and Mg^2+^ concentration of the transcription reaction. Despite assay limitations, we conclude that HIV-1_BH10_ and HIV-1_ESP49_ RTs are less accurate when copying DNA templates than RNA templates. Analysis of the RNA-dependent mutational spectra revealed a higher tendency to introduce large deletions at the initiation of reverse transcription by all HIV-1 RTs except the double-mutant K65R/V75I.

## Introduction

In retrovirus, the reverse transcriptase (RT) is the DNA polymerase responsible for the replication of the viral genome. Retroviral RTs use the (+) single-stranded RNA genome to synthesize a complementary minus-strand DNA, while the RNA template is being degraded by the RNase H activity of the RT. The newly synthesized complementary DNA (cDNA) is then used as template for the synthesis of plus-strand DNA to obtain a double-stranded proviral DNA^1,2^.

RTs are widely used in biotechnology for their ability to synthesize DNA using RNA templates. Advances introduced in the late 1990s such as cDNA microarrays and next-generation sequencing technologies have opened new possibilities for the identification of all RNA molecules in one cell or a population of cells, and the analysis of their expression levels^3–5^. RTs play a fundamental role behind these developments, and wild-type (WT) and engineered RT variants of avian myeloblastosis virus (AMV), murine leukaemia virus (MLV), human immunodeficiency virus type 1 (HIV-1) and *Geobacillus stearothermophilus* group II introns have been developed into more efficient tools to study gene expression by increasing catalytic efficiency, processivity, thermostability or fidelity of DNA synthesis^6–9^.

Unlike eukaryotic replicative polymerases, RTs lack exonuclease activity and are error-prone. Improvements in their intrinsic fidelity of DNA synthesis may have a positive impact on the reliability of whole transcriptome shotgun sequencing (i.e. RNA-seq) data^10^. Despite the large amount of research on the fidelity of retroviral RTs, most of the available studies have been devoted to the analysis of DNA-dependent DNA synthesis accuracy^11,12^. These studies have shown that oncoretroviral RTs (e.g. AMV and MLV RTs) are more faithful than lentiviral RTs such as the HIV-1 RT. Thus, the intrinsic error rates of HIV-1 RT determined with the M13mp2 *lacZα* forward mutation assay^13^ were more than 10-fold higher than those obtained with AMV and MLV RTs^14–16^. However, in HIV-1 RTs, antiretroviral drug resistance-associated mutations such as K65R or the combination of K65R and V75I were shown to increase fidelity of DNA-dependent DNA synthesis to levels similar to those obtained with oncoretroviral RTs^17,18^.

The fidelity of RNA-dependent DNA synthesis of retroviral RTs has been evaluated in enzymatic assays by comparing kinetic parameters for the incorporation of correct and incorrect nucleotides, and the extension of matched and mismatched template-primers. Assays carried out under steady-state conditions with AMV, MLV and HIV-1 RTs did not reveal large differences when DNA templates were substituted by RNA templates^19,20^. However, using pre-steady-state kinetics, Kerr and Anderson^21^ showed that misinsertion fidelity was 9–64 times (with duplex 45/25mer) and 14–23 times (with duplex 45/22mer) higher in RNA-templated than in DNA-templated reactions catalyzed by HIV-1 RT. These experiments were performed with synthetic duplexes bearing the same nucleotide sequences (except for having U instead of T in the RNA templates). Despite providing important mechanistic information, nucleotide incorporation assays are restricted to a few template-primers and therefore provide limited information on the propensity of RTs to introduce base substitutions.

In contrast, forward mutation assays based on the expression of target genes using phages such as M13 or ΦX174 give error rate estimates over a wide range of mutational sites. Attempts to compare the fidelity of retroviral RTs in DNA polymerization reactions carried out with RNA or DNA templates using these methods did not provide consistent results. Studies performed with HIV-1 RT and the amber16 reversion assay using phage ФX174 showed that two out of the seven specific mismatches analysed had 20- and 7-fold lower mutation frequencies with DNA templates than with RNA templates^22^. On the other hand, M13-based assays using *lac*Zα or the HIV-1 *env* hypervariable region 1 (V1) as target genes showed little differences in fidelity between reactions carried out with HIV-1 RT using RNA or DNA templates. Thus, base substitution error rates determined with *lacZα* were 1.7 × 10^−4^ and 1.4 × 10^−4^ for DNA-dependent and RNA-dependent reactions, respectively^23^. These small differences were also found for overall error rates in assays using a fragment of *env* as a target sequence (i.e. 1.9 × 10^−4^ with DNA templates and 2.0 × 10^−4^ with RNA templates)^24^. In contrast, and using a modified version of the M13mp2 *lac*Zα forward mutation assay, Boyer *et al*.^25^ showed that the overall fidelity of HIV-1 RT was about two to six-fold higher while copying RNA templates than DNA templates. In these experiments, authors suggested the contribution of errors made by the T7 RNA polymerase (RNAP) while preparing the RNA used as template in the fidelity assays, acknowledging an undetermined impact on the calculated error rates.

The discrepancies in the fidelity assessments could be explained in part by methodological differences in the assays, partly due to the different WT HIV-1 group M/subtype B RTs used in these experiments [i.e., homodimers (p66/p66) *versus* heterodimers (p66/p51), or RT variants derived from different viral strains, such as NL4-3, HXB2, BH10 or NY5]. In the present study, we have determined the intrinsic fidelity of RNA-dependent DNA synthesis of several RTs that showed diverse error rates in DNA-dependent DNA polymerization assays, ranging from around 1.4 × 10^−4^ in the case of HIV-1_BH10_ RT^26–29^ to 1.2 × 10^−5^ for MLV RT or ~6.3 × 10^−6^ for HIV-1 group O (ESP49 strain) RT mutants K65R (O_K65R) and K65R/V75I (O_K65R/V75I)^18^. We demonstrate how errors made by the T7 RNAP attenuate those differences while hampering an accurate determination of fidelity using RNA templates, despite improving the quality of the RNA template by using transcription conditions that could increase the accuracy of the RNA synthesis reaction. Despite those limitations, our results show that WT HIV-1_BH10_ and HIV-1_ESP49_ are less accurate when copying DNA than RNA templates.

## Results

### Fidelity of RNA-dependent DNA synthesis of retroviral RTs

M13mp2 *lacZα* forward mutation assays provide a broad estimate of the fidelity of RTs, based on a relatively large number of mutational sites and sequence contexts, although silent mutations cannot be detected by using this method. We determined mutant frequencies and error rates of RNA-dependent DNA synthesis for WT MLV, AMV, HIV-1_BH10_ and HIV-1_ESP49_ RTs, as well as mutant RTs O_K65R and O_K65R/V75I. For this purpose, a commercial T7 RNA polymerase (RNAP) was used to synthesize a *lac*Zα RNA template that was then reverse transcribed by retroviral RTs (the method is outlined in the Supplementary Fig. S1). The cDNA product was hybridised with a gapped M13mp2 DNA lacking the *lac*Zα gene in one of the two strands of the molecule. Errors made by RTs during reverse transcription result in a decrease in α-complementation and could be detected by the altered colour phenotype of the mutant plaques (pale blue or colourless) when phages are grown on an appropriated indicator strain. Mutant frequencies obtained with the six studied RTs, and calculated as the ratio of mutant to total plaques, are given in Table 1. These assays showed less than two-fold differences in fidelity between the most accurate and the least faithful RTs.

**Table 1:** RNA-dependent DNA synthesis fidelity of WT and mutant RTs in M13mp2 *lac*Zα forward mutation assays.

RTs	Mutant plaques	Total plaques	Mutant frequency^a^
HIV-1_BH10_	52	12,836	0.00405
HIV-1_ESP49_	46	13,347	0.00345 (1.18)
O_K65R	54	18,284	0.00295 (1.37)
O_K65R/V75I	69	23,512	0.00293 (1.38)
MLV	36	13,569	0.00265 (1.53)
AMV	28	11,250	0.00249 (1.63)

For each enzyme, mutant plaques were obtained after transfection of gapped DNA hybridised with the cDNA product of ten synthesis reactions. The RNA used as template in the reverse transcription reaction was synthesized by the T7 RNAP (Promega) in a transcription buffer containing 40 mM Tris-HCl pH 7.9 and 6 mM MgCl_2_ (full composition given in Materials and Methods).

^a^Background frequencies in these assays were estimated to be less than one in 20,000 plaques^18^. Numbers between parentheses indicate the fold-increase in fidelity relative to the WT HIV-1_BH10_ RT.

The mutational specificity of the studied RTs was determined after sequencing the *lac*Zα mutants generated with the M13mp2-based mutation assays (Supplementary Figs S2–S7). All mutational spectra had insertions of one thymidine at the homopolymeric sequence located at positions +137/+139. In addition, U-to-C transitions at positions −36/−35 and U-to-A transversions at position +73 were observed in the spectra of five RTs. On the other hand, we also found large deletions in all mutational spectra, except in the one generated with the AMV RT. In the case of HIV-1_BH10_, HIV-1_ESP49_ and the O_K65R RTs, the large deletions clustered at positions +172/+173 (Supplementary Figs S2–S4), corresponding to the first two nucleotides incorporated during reverse transcription. These deletions could derive from misalignment errors occurring while extending the DNA primer.

Despite the similarities found among the mutational spectra, there were also remarkable differences. Thus, for example, AMV RT generated one-nucleotide insertions of A in the homopolymeric region located at positions +91/+94 (Supplementary Fig. S7), whereas MLV RT showed one hotspot at position +144, including various deletions and transversions (A-to-C and A-to-T) (Supplementary Fig. S6). In our analyses, hotspots were defined as those positions where at least four mutations were found. The statistical differences in hotspot distribution between different RTs were determined by using a two-tailed Fisher’s exact test, and are shown in the Supplementary Table S1. Interestingly, the spectrum of the O_K65R/V75I RT showed an important hotspot at position +147 (U-to-C substitutions) (Supplementary Fig. S5). This hotspot is absent in the mutational spectra of the other analysed RTs (*P* < 0.005, for all five comparisons) (Supplementary Table S1). On the other hand, mutations induced by HIV-1_ESP49_ RT seemed to be scattered throughout the whole *lacZα* sequence (Supplementary Fig. S3). The spectrum induced by the single-mutant O_K65R had one hotspot at position +109 (Supplementary Fig. S4), while in the one obtained with HIV-1_BH10_ RT, G-to-T changes accumulated at position +149 (Supplementary Fig. S2).

Error rates for all RTs are summarized in Table 2. The highest error rates were obtained with WT HIV-1_BH10_ RT, while one of the most faithful enzymes was the double-mutant O_K65R/V75I that showed 1.4-fold increased accuracy relative to the HIV-1_BH10_ RT. Mutant O_K65R/V75I RT had a low tendency to introduce frameshifts, although it had a relatively high base substitution error rate. This enzyme was prone to generate transitions (81%), which accumulated at two major hotspots (at positions −36 and +147) and three minor hotspots (located at nucleotides −35, −7 and +87). Unlike in the case of O_K65R/V75I, this strong bias towards the generation of transitions was not observed with the other RTs studied. On the other hand, compared with the other RTs, the HIV-1_BH10_ polymerase was prone to introduce frameshift errors, which were predominantly deletions.

**Table 2:** Summary of error rates for WT and mutant RTs, for various classes of mutations in M13mp2 *lac*Zα forward mutation assays.

Mutation type	HIV-1_BH10_ RT		AMV RT		MLV RT		HIV-1_ESP49_ RT		O_K65R RT		O_K65R/V75I RT	
	No. of errors	Error rate	No. of errors	Error rate	No. of errors	Error rate	No. of errors	Error rate	No. of errors	Error rate	No. of errors	Error rate
**All classes**	**54**	**1/28524**	**43**	**1/31395**	**46**	**1/35397**	**44** ^a^	**1/36401**	**58**	**1/37829**	**71**	**1/39739**
**Base substitutions**	**26**	**1/36434**	**25**	**1/33210**	**29**	**1/34531**	**19**	**1/51843**	**29**	**1/46530**	**53**	**1/32739**
Transitions	11 (42%)		10 (40%)		12 (41%)		10 (53%)		19 (66%)		43 (81%)	
Transversions	15 (58%)		15 (60%)		17 (59%)		9 (47%)		10 (34%)		10 (19%)	
**Frameshifts**	**28**	**1/48960**	**18**	**1/66750**	**17**	**1/85245**	**25**	**1/57018**	**29**	**1/67336**	**18**	**1/139505**
Insertions	7 (25%)		14 (78%)		9 (53%)		13 (52%)		10 (34%)		9 (50%)	
Deletions	21 (75%)		4 (22%)		8 (47%)		12 (48%)		19 (66%)		9 (50%)	
At runs^b^	10 (36%)	1/29266	10 (56%)	1/25650	7 (41%)	1/44196	12 (48%)	1/25359	13 (45%)	1/32067	12 (67%)	1/44673
At non-runs	18 (64%)	1/59901	8 (44%)	1/118125	10 (59%)	1/113980	13 (52%)	1/86242	16 (55%)	1/95991	6 (33%)	1/329168

The RNA used as template in the reverse transcription reaction was synthesized by the T7 RNAP (Promega) in a transcription buffer containing 40 mM Tris-HCl pH 7.9 and 6 mM MgCl_2_.

^a^The total number of errors with this RT was smaller than the number of mutant plaques (Table 1), because five of those mutant plaques were either recombinants or had complex arrays of mutations, and were not included in the analysis of error rates.

^b^A run is considered when there is a row of three or more identical nucleotides.

By using the forward mutation assay we cannot determine whether the errors were made by the T7 RNAP during the synthesis of the RNA template (transcription) or by RTs during cDNA synthesis (reverse transcription). The overall error rates obtained with the RTs used in this study were in the range of 2.5 × 10^−5^ to 3.5 × 10^−5^. These values are relatively close to reported estimates of transcription error rates obtained in different organisms^30–33^, and suggest the existence of an inaccuracy threshold imposed by the T7 RNAP while synthesizing the RNA template.

### Fidelity of promoter-dependent transcription by T7 RNA polymerase

In order to reduce the possible inaccuracy threshold imposed by the T7 RNAP in M13mp2-based assays, we analysed its fidelity under different reaction conditions. A linearized pTRI-β-actin-Mouse plasmid containing a T7 promoter sequence was used as template for *in vitro* transcription using T7 RNAP (Ambion) in the presence of [α-^32^P]CTP. Reactions were carried out at different pH and Mg^2+^ concentrations, either in the presence of all four NTPs or with biased NTP pools (i.e. lacking or with a very low concentration of one ribonucleotide) (Supplementary Fig. S8). In this assay, when all NTPs were present (lanes marked with an asterisk), the polymerase synthesized an RNA of 53 nucleotides. When using biased pools, the amount of full-length RNA was reduced, since the insertion of incorrect nucleotides would be needed to complete the synthesis reaction.

Relative amounts of full-length products in reactions carried out with biased NTP mixtures and in the presence of all NTPs provided a rough estimate of the accuracy of T7 RNAP in different assay conditions. It should be noted that G is required to initiate transcription, and therefore T7 RNAP cannot synthesize RNA in the absence of this nucleotide (lanes 3 in Supplementary Fig. S8). Inefficient RNA synthesis was also observed in the absence of A or U (lanes 1 and 6). When ATP was supplied at 1 µM while maintaining the three other NTPs at 100 µM (lanes 2), a small but significant amount of full-length RNA was observed at higher magnesium concentrations, as well as above pH 7.0. Similar findings were obtained in reactions with NTP mixtures having low concentrations of GTP (10–20 µM) (lanes 4 and 5). The amount of full-length products in biased reactions was greater at 6 mM MgCl_2_ and at pH 7.5, suggesting a lower accuracy in those conditions. At pH 5.5–6.0 we did not detect any transcription products. In addition, the efficiency of the reaction was very low in the presence of 0.5 mM MgCl_2_. Taken together, our data indicate that T7 RNAP showed good activity and improved accuracy at pH 6.5–7.0, and in the presence of Mg^2+^ at 1–3 mM.

### Nucleotide incorporation kinetics and template-primer binding affinity of T7 RNA polymerase

RNA templates used in forward mutation assays were synthesized under optimal conditions for transcription by T7 RNAP^34^. These reactions were carried out in 40 mM Tris-HCl buffer pH 7.9, containing 6 mM MgCl_2_. However, promoter-dependent transcription assays revealed qualitative differences in fidelity when RNA was synthesized at lower pH and in the presence of reduced amounts of magnesium. These observations indicated that transcriptions could be more accurate when carried out in buffers containing 40 mM Bis-Tris pH 6.75 and 1.5 mM MgCl_2_.

**Figure 1:**
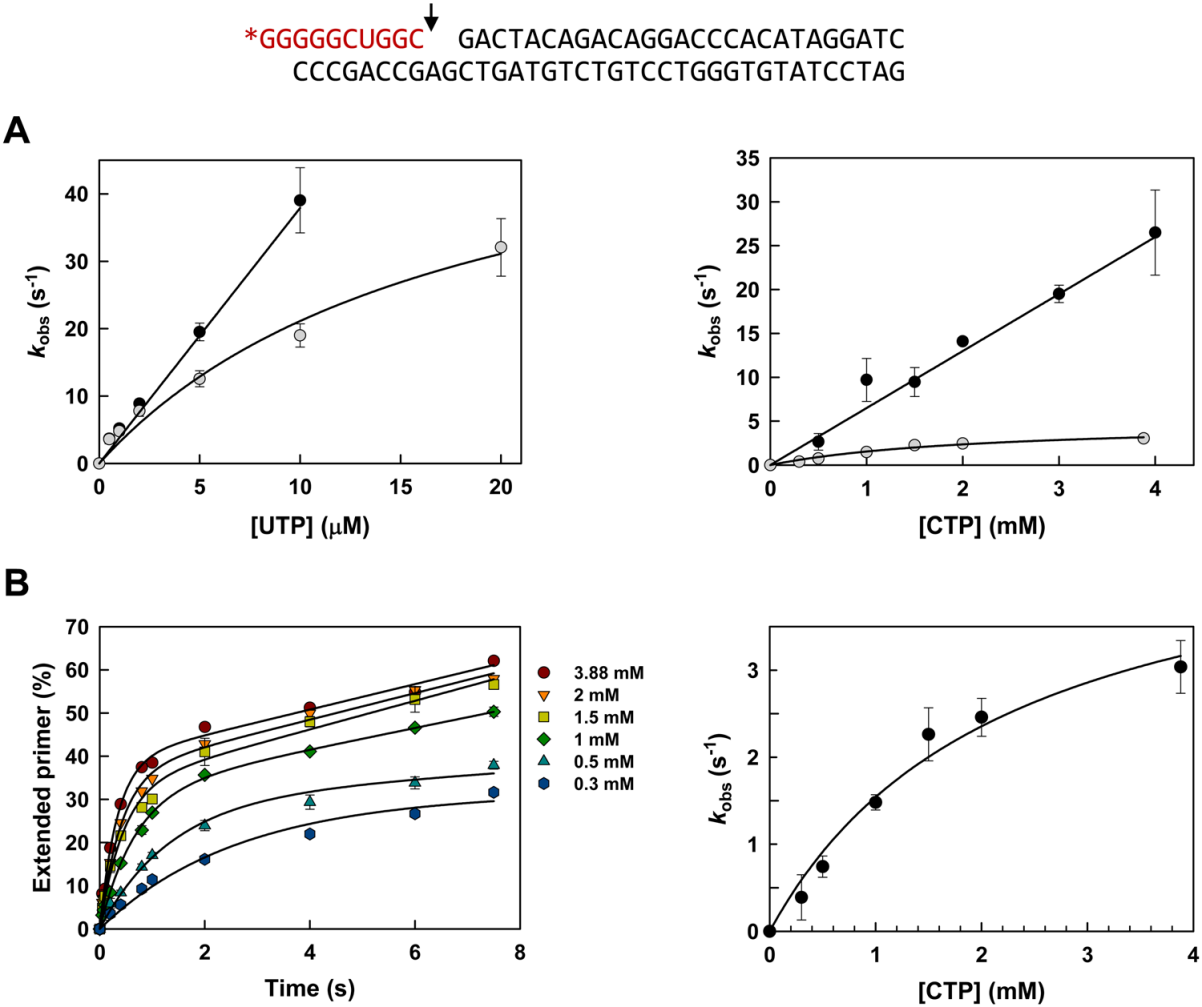
Nucleotide incorporation kinetics for UTP and CTP into VSR10 template-primer by recombinant T7 RNA polymerase. Nucleotide sequences of the VSR10 complex^56^ are shown above. VSR10 is made of a double-stranded DNA (black) annealed to a 10-nt RNA primer (red). The RNA is labelled at its 5′ end with [γ-^32^P]ATP. The arrow indicates the nucleotide incorporation site. (**A**) UTP and CTP incorporation rates at different nucleotide concentrations, obtained at pH 7.9 and 6 mM MgCl_2_ (black circles), and at pH 6.75 and 1.5 mM MgCl_2_ (grey circles). (**B**) Pre-steady-state kinetics of CTP incorporation on VSR10 by T7 RNAP, at pH 6.75 (Bis-Tris buffer) and 1.5 mM MgCl_2_. In the left panel, continuous lines represent the best fit of the data to the single-exponential equation, obtained at different nucleotide concentrations. The right panel shows the nucleotide concentration dependence of CTP incorporation. The continuous line represents the best fit of the *k*_obs_ data to the Michaelis-Menten equation. The obtained polymerization rates (*k*_pol_) and apparent equilibrium dissociation constant (*K*_d_) of CTP were 4.98 ± 0.71 s^−1^ and 2.23 ± 0.61 mM, respectively. Results were obtained from three independent experiments.

The efficiency and nucleotide specificity of the T7 RNAP was determined in single-nucleotide incorporation assays using a VSR10 hybrid containing a dsDNA and a 10-nucleotide RNA primer (Fig. 1). Due to the requirement of relatively large amounts of enzyme, these assays were carried out with purified recombinant T7 RNAP. Nucleotide incorporation rates were determined at different concentrations of correct (UTP) or incorrect ribonucleotides (ATP, CTP and GTP) in standard buffer conditions (40 mM Tris-HCl pH 7.9, containing 6 mM MgCl_2_), as well as in conditions enhancing T7 RNAP fidelity (40 mM Bis-Tris buffer pH 6.75 and 1.5 mM MgCl_2_). Kinetic constants for UTP incorporation could not be obtained due to the high efficiency of the polymerization reaction. Thus, at nucleotide concentrations above 300 µM, the T7 RNAP showed UTP incorporation rates (*k*_obs_) above 150 s^−1^ in both assay conditions. At low concentrations of UTP, the polymerase showed different kinetic behaviours at pH 7.9/6 mM Mg^2+^ and pH 6.75/1.5 mM Mg^2+^ (Fig. 1A). The relationship between *k*_obs_ and [UTP] was linear at pH 7.9/6 mM Mg^2+^ but hyperbolic at pH 6.75/1.5 mM Mg^2+^. Similar kinetics were observed in CTP incorporation reactions, although nucleotide incorporation efficiencies were much lower at pH 6.75/1.5 mM Mg^2+^ than in reactions carried out in 40 mM Tris-HCl buffer pH 7.9, containing 6 mM MgCl_2_. These data revealed that relative incorporation rates of CTP (incorrect) *versus* UTP (correct) were lower at pH 6.75/1.5 mM Mg^2+^ than at pH 7.9/6 mM Mg^2+^. However, reliable estimates of kinetic constants were obtained only for the misincorporation of C at pH 6.75/1.5 mM Mg^2+^ (Fig. 1B).

Unlike in the case of pyrimidines, the efficiency of ATP and GTP misincorporation by T7 RNAP was much lower. Kinetic parameters, given in the Supplementary Table S2, showed that T7 RNAP incorporated non-complementary purines with 2.6- to 3.0-fold higher efficiencies at pH 7.9/6 mM Mg^2+^ than at pH 6.75/1.5 mM Mg^2+^. ATP was incorporated more efficiently than GTP in both reaction conditions. The catalytic efficiency of ATP incorporation was 29.15 ± 15.17 M^−1^s^−1^ at pH 7.9/6 mM Mg^2+^ and 11.05 ± 1.53 M^−1^s^−1^ at pH 6.75/1.5 mM Mg^2+^. Similar differences were obtained for GTP, although at pH 6.75/1.5 mM the *k*_pol_/*K*_d_ of the T7 RNAP was only 6.60 ± 1.16 M^−1^s^−1^. Taken together, these data provide further support to the notion that at pH 6.75 and 1.5 mM Mg^2+^ the T7 RNAP showed a modest but consistent improvement in its fidelity of RNA synthesis.

**Figure 2:**
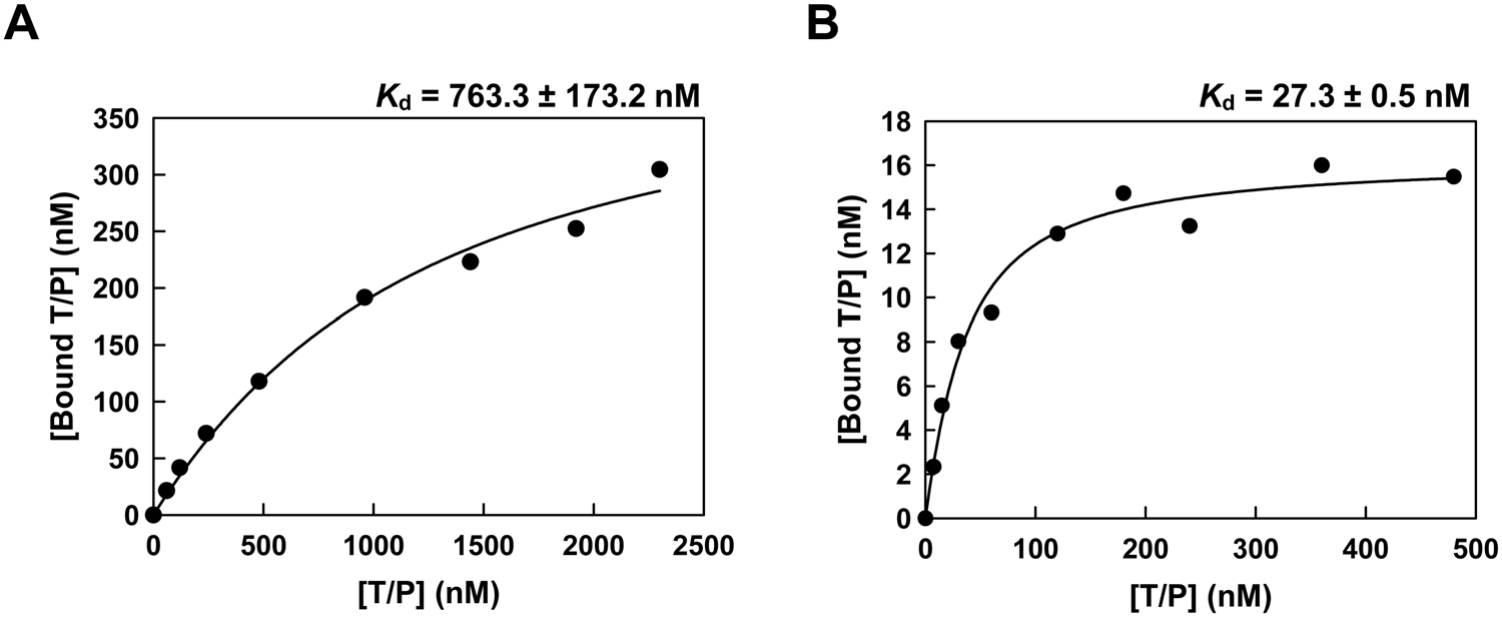
Dissociation equilibrium constants (*K*_d_) for T7 RNA polymerase and the VSR10 dsDNA/RNA heteroduplex. Data shown were obtained from representative assays carried out at pH 7.9 and 6 mM MgCl_2_ (panel A) and at pH 6.75 and 1.5 mM MgCl_2_ (panel B). The solid line represents the best fit of the data to the quadratic equation relating the template-primer bound to the T7 RNAP and the total concentration of the VSR10 heteroduplex. Reported *K*_d_ values represent averages ± standard deviations obtained from at least three independent experiments.

Interestingly, in addition to its effects on fidelity, the assay conditions had a strong influence on template-primer binding. At pH 7.9 and 6 mM Mg^2+^, the dissociation equilibrium constant (*K*_d_) for T7 RNAP and the VSR10 heteroduplex was about 30 times higher than at pH 6.75 and 1.5 mM MgCl_2_ (Fig. 2). The higher affinity for VSR10 of the T7 RNAP at lower pH and Mg^2+^ concentration could represent an additional advantage towards increasing the efficiency of RNA synthesis in the presence of limiting amounts of template.

### Estimates of RNA-dependent DNA synthesis fidelity of HIV-1 RTs using more faithful RNA templates

Although the biochemical studies reveal that it is possible to increase transcription fidelity by reducing the pH and magnesium concentration of the reaction, it is not clear whether this would affect estimates of fidelity of RNA-dependent DNA synthesis of retroviral RTs. As shown in Tables 1 and 2, the highest mutant frequency and error rate obtained with RNA templates synthesized at pH 7.9 and in the presence of 6 mM MgCl_2_ were found in assays carried out with the HIV-1_BH10_ RT. We used this enzyme to test the impact of the RNA template on mutant frequencies obtained with M13mp2 *lacZα* forward mutation assays. Theoretically, more faithful RNAs (i.e. those carrying less transcription errors) would reduce the mutational threshold of the assay. RNAs containing the *lacZα* sequence were synthesized using recombinant T7 RNAP in the two different conditions (i.e. pH 7.9/6 mM Mg^2+^ and pH 6.75/1.5 mM Mg^2+^). Lower mutant frequencies were obtained with HIV-1_BH10_ RT using RNA templates synthesized at lower pH and Mg^2+^ concentrations, although the differences were not large (Table 3). The analysis of the mutational spectra showed similar distributions of transitions and transversions using both RNAs, but at the lower pH and Mg^2+^ concentration, we observed an increased tendency to introduce frameshift errors in homopolymeric *versus* heteropolymeric sequences (Supplementary Figs S9 and S10). In any case, base substitution and frameshift error rates were about 49–58% lower in assays carried out with RNA obtained using the pH 6.75/1.5 mM Mg^2+^ conditions (Table 4).

**Table 3:** RNA-dependent DNA synthesis fidelity of HIV-1 RTs in M13mp2 *lac*Zα forward mutation assays.

RTs	T7 RNAP source	Transcription conditions		Mutant plaques	Total plaques	Mutant frequency
		pH	[Mg^2+^] (mM)			
HIV-1_BH10_	Recombinant	7.9	6	45	4,650	0.00968
HIV-1_BH10_	Recombinant	6.75	1.5	70	11,226	0.00624
O_K65R/V75I	Recombinant	6.75	1.5	56	14,735	0.00380
O_K65R/V75I	Commercial	6.75^a^	1.5	51	19,466	0.00262

Template RNA used in these assays was obtained in T7 RNAP-catalyzed reactions carried out at different pH and [Mg^2+^], as indicated.

^a^This assay was carried out with the T7 RNAP from Promega in PIPES buffer (pH 6.75), and results were obtained after pooling the products of six reverse transcription reactions.

**Table 4:** Summary of error rates for RNA-dependent DNA synthesis catalyzed by HIV-1 RTs, and obtained with RNAs synthesized by the T7 RNAP under different assay conditions.

Mutation type	HIV-1_BH10_ RT				O_K65R/V75I RT			
	Recombinant T7 RNAP (pH 7.9; 6 mM MgCl_2_)		Recombinant T7 RNAP (pH 6.75; 1.5 mM MgCl_2_)		Recombinant T7 RNAP (pH 6.75; 1.5 mM MgCl_2_)		Commercial T7 RNAP ^a^ (pH 6.75; 1.5 mM MgCl_2_)	
	No. of errors	Error rate	No. of errors	Error rate	No. of errors	Error rate	No. of errors	Error rate
**All classes**	**47**	**1/11872**	**74** ^b^	**1/18204**	**59**	**1/29969**	**53**	**1/44074**
**Base substitutions**	**26**	**1/13199**	**42**	**1/19726**	**40**	**1/27186**	**32**	**1/44893**
Transitions	14 (54%)		25 (60%)		30 (75%)		19 (59%)	
Transversions	12 (46%)		17 (40%)		10 (25%)		13 (41%)	
**Frameshifts**	**21**	**1/23649**	**32**	**1/37467**	**19**	**1/82826**	**21**	**1/98999**
Insertions	10 (48%)		19 (59%)		16 (84%)		9 (43%)	
Deletions	11 (52%)		13 (41%)		3 (16%)		12 (57%)	
At runs ^c^	10 (48%)	1/10602	21 (66%)	1/12188	15 (79%)	1/22397	11 (52%)	1/40348
At non-runs	11 (52%)	1/35509	11 (34%)	1/85726	4 (21%)	1/309435	10 (48%)	1/163514

^a^The T7 RNAP used in these assays was obtained from Promega, and buffers for the corresponding RNA synthesis reactions were prepared with PIPES.

^b^Recombinant mutants are excluded from the analysis.

^c^A run is considered when there is a row of three or more identical nucleotides.

In previous studies, we found that the mutant O_K65R/V75I RT was >15-fold more accurate than the HIV-1_BH10_ RT in forward mutation assays measuring fidelity of DNA-dependent DNA synthesis^18,29^. Although mutant frequencies obtained with the double-mutant RT using RNAs synthesized at pH 6.75 and in the presence of 1.5 mM MgCl_2_ were lower than those obtained with HIV-1_BH10_ RT (Table 3), the effects were not large. Error rates of 3.3 × 10^−5^ were estimated for the O_K65R/V75I RT under those conditions (Table 4). Interestingly, the analysis of mutational spectra revealed that major hotspots obtained with O_K65R/V75I RT while reverse transcribing an RNA template synthesized at pH 7.9/6 mM Mg^2+^ (Supplementary Fig. S5) were absent from the spectra obtained with the RNA made at pH 6.75/1.5 mM Mg^2+^ (Supplementary Fig. S11). Examples of hotspots that seemed to be lost with RNAs obtained at lower pH and Mg^2+^ concentration included clusters of U-to-C transitions found at positions −36, −35 and +147.

The analysis of data shown in Tables 1–4 also revealed that RNA synthesized by recombinant T7 RNAP had lower quality than the RNA made with commercial preparations of the enzyme that rendered lower mutant frequencies in M13mp2-based assays, even at pH 7.9 and high Mg^2+^ concentration. Thus, error rates obtained with the HIV-1_BH10_ RT using RNA synthesized at pH 7.9/6 mM Mg^2+^ were estimated to be 8.4 × 10^−5^ with the recombinant T7 RNAP and 3.5 × 10^−5^ with the commercial enzyme. Similar experiments with O_K65R/V75I RT and RNAs transcribed at pH 6.75/1.5 mM Mg^2+^ showed error rates of 3.3 × 10^−5^ and 2.3 × 10^−5^ for recombinant and commercial T7 RNAPs, respectively.

The smaller differences observed with the O_K65R/V75I RT relative to the HIV-1_BH10_ RT can be attributed to the existence of a transcriptional threshold that has a bigger influence on more faithful RTs. This limit seems to be achieved with RNAs obtained at pH 6.75/1.5 mM Mg^2+^ with the commercial T7 RNAP. However, when the commercial enzyme is used, the pH and Mg^2+^ concentration had a relatively small influence in the error rate (2.5 × 10^−5^, with RNAs obtained at pH 7.9/6 mM Mg^2+^) while differences may not be significant. Interestingly, and in agreement with our proposal, the error rate of HIV-1_BH10_ RT was reduced by 1.6-fold when the RNAs were synthesized by recombinant T7 RNAP at pH 6.75/1.5 mM Mg^2+^, as compared with those obtained at pH 7.9/6 mM Mg^2+^. Meanwhile, the smaller reduction in the error rate (1.1-fold) observed with O_K65R/V75I RT when changing transcription conditions might be attributed to the use of commercial T7 RNAP to synthesize the RNA templates.

The origin of the T7 RNAP had a minor effect on hotspot distribution in the O_K65R/V75I spectra when the template RNA was synthesized at lower pH and magnesium concentration (Supplementary Figs S11 and S12). Thus, major hotspots at positions +87 and +139 were found with RNAs synthesized at pH 6.75/1.5 mM Mg^2+^, with recombinant or commercial T7 RNAP. Differences in hotspot distribution along the *lac*Zα sequence between both assays were not significant statistically.

## Discussion

**Figure 3:**
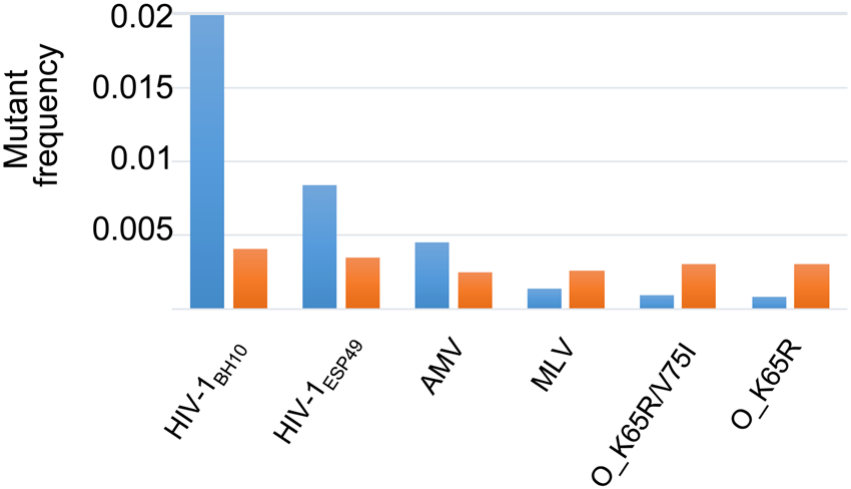
Comparison of RNA- and DNA-dependent DNA synthesis fidelities of retroviral RTs. Estimates of fidelity of RNA-dependent DNA synthesis are based on mutant frequencies obtained with the adapted M13mp2 *lacZα* forward mutation assay, reported in this paper (orange bars). Blue bars represent previously reported values, obtained using M13mp2-based assays measuring the accuracy of DNA-dependent DNA synthesis of HIV-1_BH10_^29^, HIV-1_ESP49_^36^, AMV^14^, and MLV, O_K65R/V75I and O_K65R RTs^18^.

Previous studies showed large variations in estimates of fidelity of retroviral RTs while synthesizing DNA using DNA templates (reviews^12,35^). The results obtained in the present study show that the intrinsic fidelity of RNA-dependent DNA synthesis varies within the range of 2.3 × 10^−5^ to 5.5 × 10^−5^, in assays carried out with RNA transcribed by T7 RNAP at pH 6.75 and in the presence of 1.5 mM Mg^2+^. This narrow window of variation was also observed with RNA templates obtained in conditions where the T7 RNAP was found to be less faithful (i.e. pH 7.9/6 mM Mg^2+^), rendering overall error rates of 2.5 × 10^−5^ to 8.4 × 10^−5^. The comparison of mutant frequencies obtained with M13mp2 *lacZα* forward mutation assays using DNA or RNA templates (Fig. 3) illustrates these differences. While HIV-1_BH10_ RT is >15-fold less accurate than HIV-1_ESP49_ mutant RTs K65R and K65R/V75I in DNA-dependent DNA synthesis reactions, differences between the most and least faithful RTs fall within less than two-fold in assays carried out with RNA templates (Table 1). Nonetheless, HIV-1_BH10_ RT remains as the least faithful enzyme in both types of assays. All the results reported in Fig. 3 were obtained in our lab following the same experimental procedures, except in the case of mutation frequencies determined for AMV RT with DNA-dependent DNA synthesis fidelity assays. Our previous studies measuring the fidelity of DNA-dependent DNA synthesis have shown that the mutant frequency variability obtained in forward mutation assays was under 30% for HIV-1_BH10_ and HIV-1_ESP49_ RTs^28,29,36^, in agreement with values reported by other laboratories using mutant and WT RTs^37,38^.

Intrinsic error rates of RTs in DNA-dependent DNA synthesis can be as low as 6.3 × 10^−6^ (e.g. for O_K65R and O_K65R/V75I RTs)^18^, with RNA templates we obtained error rates that were more than three-fold higher with all tested enzymes. Structural studies with HIV-1 RT bound to RNA/DNA template-primers^39–41^ have shown that the RT adopts a similar conformation than in complexes containing DNA/DNA hybrids. However, in the structures containing RNA/DNA complexes, there are additional contacts involving residues of the p51 subunit as well as 2′-OH groups of the RNA template. These interactions contribute to the higher affinity of RT for RNA/DNA hybrids reported in biochemical studies^42^. Although differences in the interaction with RNA/DNA complexes *versus* DNA/DNA template-primers could be potentially responsible for the different error rates obtained in assays measuring fidelity of RNA- and DNA-dependent DNA synthesis, a more likely possibility is that those differences are due to transcription errors affecting the quality of the RNA template used in the assays.

Transcription errors in prokaryotic and eukaryotic cells are very difficult to quantify and large differences in error rates have been reported by several groups. Nonetheless, rough estimates of around 10^−5^ per nucleotide have been reported^30–32,43^. Moreover, in a recent study, Traverse and Ochman^33^ found conserved transcription error rates (ranging from 2.3 × 10^−5^ to 5.2 × 10^−5^ per nucleotide) across different species of bacteria, determined under different growth conditions and by considering either messenger or ribosomal RNA sequences. Those values were consistent with error rates obtained in our study, suggesting that T7 RNAP might be responsible for the inaccuracy threshold observed in our assays. Unfortunately, we are not aware of any reliable estimates of fidelity for T7 RNAP, although misincorporation rates have been estimated around 5 × 10^−5^ ^44^, in good agreement with average base substitution error rates of 3 × 10^−5^ determined by Remington *et al*.^45^ with a codon reversion assay.

Our results also show that the accuracy of the T7 RNAP can be manipulated to some extent by changing transcription conditions, although reductions of the inaccuracy threshold were modest. T7 RNAP synthesizes more faithful RNA at lower pH (6.75 *vs*. 7.9) and Mg^2+^ concentration (1.5 mM *vs*. 6 mM). This increase in fidelity at lower pH conditions has also been previously reported for several DNA polymerases (e.g *Taq* DNA polymerase, exonuclease-deficient Klenow fragment of *E. coli* DNA polymerase I, human DNA polymerase α and HIV-1 RT)^46–48^. Thus, increases of fidelity of up to 50-fold have been reported at pH 6.2 compared to pH 9.8 for base substitutions using the Klenow fragment of *E. coli* DNA polymerase I^47^. Authors attributed these effects to altered template binding properties of the enzyme at a lower pH. In the case of T7 RNAP, nucleotide incorporation studies with nonpolar thymidine analogues have shown that hydrogen bonds are important for polymerization efficiency due to their role in stabilizing the closed ternary complex of the polymerase, but electrostatic interactions with the minor groove of the substrate were critical for fidelity^49^. On the other hand, it has been shown that HIV-1 RT and *Taq* DNA polymerase display higher fidelity at low concentrations of Mg^2+^ (e.g. 0.25 mM), although this property was not observed in experiments carried out with the MLV RT^46,50^. Despite improvements in fidelity obtained by reducing pH and Mg^2+^ concentrations, there are obvious limitations to this approach due to the loss of catalytic activity of the enzymes when departing from the optimal conditions for their polymerization activity.

**Figure 4:**
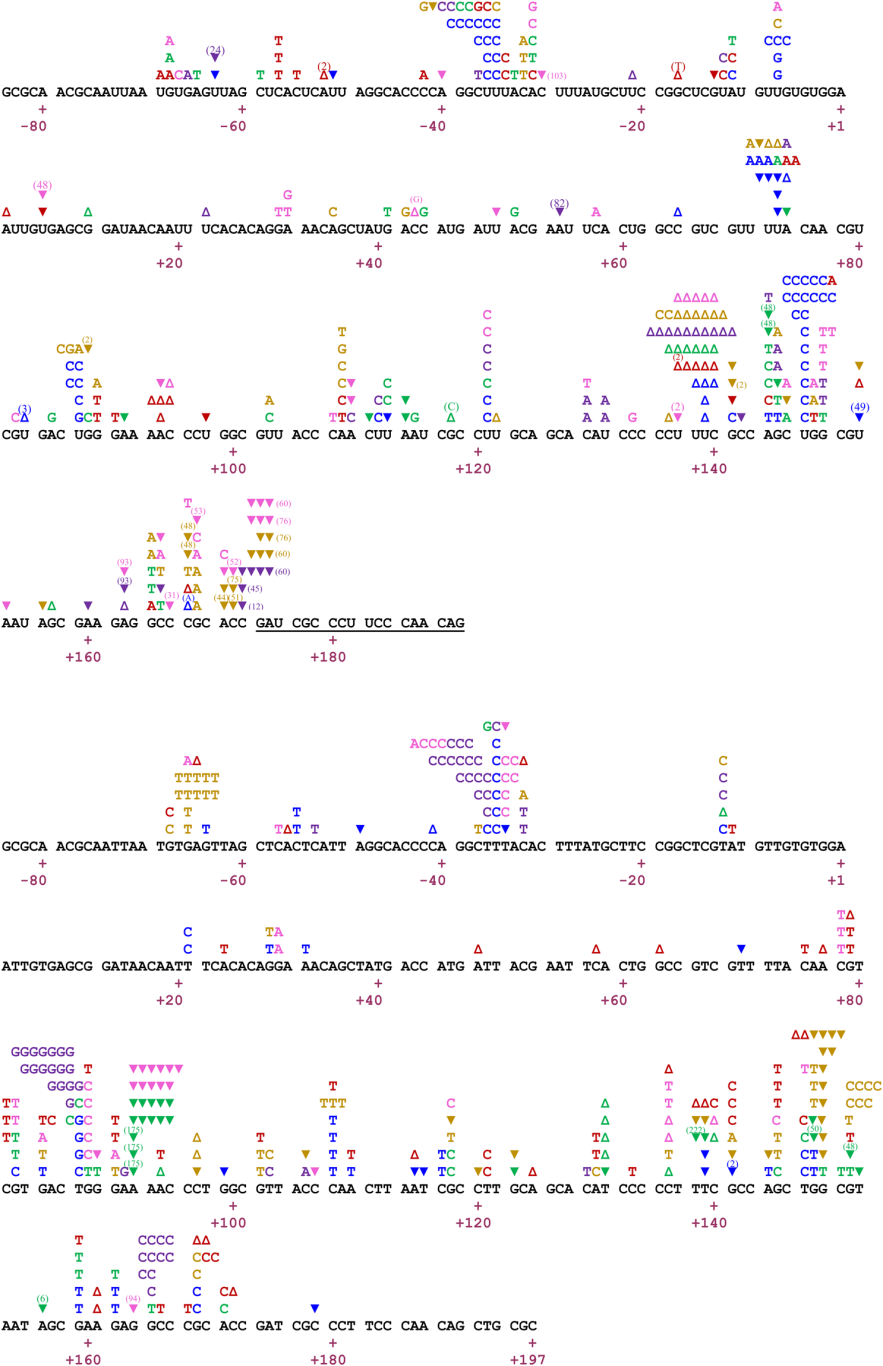
Comparison of mutational spectra induced by retroviral RTs during RNA- and DNA-dependent DNA synthesis. (Up) Combined mutational spectra derived from RNA-dependent DNA synthesis reactions including all RTs analysed in this study (Supplementary Figs S2 to S7). RTs included are those of WT HIV-1_BH10_ (pink), HIV-1_ESP49_ (purple), AMV (red), and MLV (green), and mutants O_K65R/V75I (blue) and O_K65R (brown). (Bottom) Combined mutational spectra induced during DNA-dependent DNA synthesis reactions, and taken from previously published reports^15,18,36^. RT colour codes are the same as above. For all RTs and mutational spectra, single-nucleotide substitutions are indicated by the letter corresponding to the new base above the template sequence of the *lac*Zα target. Open upright triangles represent insertions and inverted triangles indicate deletions. The triangles are positioned at the 3′ end of the frameshift, with the number of inserted/deleted nucleotides indicated between parentheses. If not specified, one-nucleotide insertions correspond to the duplication of the base where the triangle is positioned.

By analysing the mutational spectra induced by the studied RTs we also obtained relevant information on preferred hotspots and types of errors on different templates (i.e. RNA *versus* DNA), and the contribution of specific errors likely made by T7 RNAP and found in RNA templates used in the assays. The mutational spectra of the six RTs studied (gathered in Fig. 4) show that RTs are prone to introduce errors when copying RNA or DNA templates at positions −36/−34 (mostly, U-to-C or T-to-C transitions), +139/+149, and within nucleotides +165 and +171. In the latter case, transversions are largely predominant with the DNA template, while transitions, frameshift errors and large deletions dominated the RNA-dependent mutational spectra. In addition, we observed a different distribution of hotspots when comparing spectra obtained with RNA or DNA templates. The DNA-dependent mutational spectra showed scattered hotpots at positions −66, +108, +118, +136 and clustered at nucleotides +87 to +92. In contrast, the RNA-dependent spectra contained hotspots at positions −7, +73, +87, +109 and +121. Among them, the hotspot at position +73 was represented mainly by U-to-A transversions and one-nucleotide frameshifts.

The mutational spectra induced by RTs during RNA-dependent DNA synthesis contain a prominent hotspot at the homopolymeric region located at +137/+139. Errors at this position consist of insertions of one T in a run of three thymidines. These errors were previously detected by Boyer *et al*.^25^ in spectra obtained with HIV-1 and AMV RTs, and attributed to errors made by the T7 RNAP and therefore, found in the RNA template used by retroviral RTs in the cDNA synthesis reaction. Our results are consistent with that proposal, since we also found one-nucleotide insertions at this site in all of the analysed mutational spectra, without statistically significant differences between them (Supplementary Table S1). Interestingly, the insertions were not detected in the mutational spectrum induced by HIV-1 RT using *lac*Zα RNA templates synthesized with the T3 RNAP^23^, supporting the notion that this type of errors are specifically made by the T7 RNAP. Another important hotspot represented by many large deletions occurs at positions +167/+ 173, at the 3′ end of the primer used for cDNA synthesis by the retroviral RTs. Deletions were more frequent at positions that correspond to the incorporation of the first and second nucleotides (i.e. +172 and +173), and probably result from either inefficient extension of the primer during DNA synthesis or aberrant RT/template-primer interactions. Interestingly, frameshift errors near the 3′ end of the primer used in cDNA synthesis had been previously reported by Boyer *et al*.^25^, although in that study the hotspot induced by HIV-1 RT appeared at position +158. Interestingly, the authors also noted that this hotspot was absent from the mutational spectra generated with AMV RT. In our experiments, the large deletions at the 3′ end of the primer were found only in the mutational spectra induced by HIV-1_BH10_, HIV-1_ESP49_ and the O_K65R RT, but not with oncoretroviral RTs such as MLV or AMV RTs and the HIV-1_ESP49_ mutant K65R/V75I. The presence of this particular hotspot could be partly explained by the relatively high template-primer dissociation rate of HIV-1 RT during the first nucleotide incorporation events^51^.

In summary, our study further demonstrates that retroviral RTs such as HIV-1_BH10_ (group M subtype B) and HIV-1_ESP49_ (group O) are more faithful when copying RNA than when copying DNA templates. For enzymes showing higher accuracy such as AMV RT, MLV RT and the mutant HIV-1_ESP49_ RTs O_K65R and O_K65R/V75I, differences in accuracy cannot be determined in a reliable manner due to the sequence heterogeneity of the RNA used as template. Transcription errors, and specifically in our assays those made by the T7 RNAP, hamper a correct assessment of fidelity in RNA-dependent DNA synthesis reactions, even after changing the reaction conditions to improve the quality of the RNA template. Further methodological developments including a better assessment of RNA-dependent DNA synthesis fidelity of retroviral RTs are expected to be helpful for improving next-generation sequence platforms that require reverse transcription for RNA sequencing.

## Experimental Procedures

### Enzymes

Heterodimeric (p66/p51) wild-type (WT) RTs of HIV-1 group M strain BH10 (HIV-1_BH10_), HIV-1 group O strain ESP49 (HIV-1_ESP49_) and HIV-1_ESP49_ mutants K65R and K65R/V75I (O_K65R and O_K65R/V75I, respectively) were obtained as previously described^18,36,52^. RT p66 subunits with His_6_ tags at their C-terminus were co-expressed with HIV-1 protease in *Escherichia coli* XL1 Blue. RT heterodimers were purified to homogeneity by ionic exchange on cellulose phosphate P11 (Whatman), followed by affinity chromatography on Ni^2+^-nitriloacetic-agarose (ProBond^TM^ resin, Invitrogen)^53^. Purity of the enzymes was assessed by sodium dodecyl sulfate-polyacrylamide gel electrophoresis (SDS-PAGE). RT concentrations were determined spectrophotometrically using a molar extinction coefficient of 2.6 × 10^5^ M^−1^ cm^−1^ at 280 nm, and RTs were titrated to determine their active site concentration^54^. MLV and AMV RTs were obtained from Promega (catalogue #M170A and #M5101, respectively).

T7 RNA polymerase (RNAP) was obtained from commercial sources (Promega catalogue #P2075 and Ambion catalogue #AM1312), or purified after expression in *E. coli* BL21 (pREP4), using the plasmid pQE9T7 encoding the full-length enzyme with a His_6_ N-terminal extension^55^. The plasmid pQE9T7 was kindly provided by Ralf Ehricht (Alere Technologies Gmbh, Jena, Germany). Recombinant T7 RNAP was purified by affinity chromatography on Ni^2+^-nitriloacetic-agarose, as previously reported^55^. The purity of the enzyme was assessed by SDS-PAGE and its concentration was calculated from its absorbance at 280 nm and using a molar extinction coefficient of 1.4 × 10^5^ M^−1^ cm^−1^.

### Promoter-dependent transcription assays

The plasmid pTRI-β-actin-Mouse (included in the Ambion MAXIscript® T7 Transcription Kit) was used by T7 RNAP as the promoter-dependent template in transcription assays. These assays were used to assess nucleotide misincorporation efficiencies in the absence of one NTP or with unbalanced mixtures of NTPs under different transcription conditions. First, the plasmid was digested for 90 min at 37 °C with EcoRI (New England Biolabs) in 50 µl of 100 mM Tris-HCl buffer pH 7.5, containing 50 mM NaCl, 10 mM MgCl_2_, 0.025% Triton X-100 and 3 units of the enzyme per µg of plasmid. The resulting linearized plasmid was analysed in 1% agarose gels and purified with the NucleoSpin® Gel and PCR Clean-up Kit (Macherey-Nagel) according to the manufacturer’s instructions.

Transcription assays were carried out at 37 °C for 0–30 min in different buffers depending on the desired pH (i.e. MES, PIPES, Bis-Tris and Tris-HCl). Reactions were done in 20 µl of 40 mM buffer (pH 5.5–7.5), containing 10 mM NaCl, 0.5–6 mM MgCl_2_, 2 mM spermidine, 10 mM dithiothreitol (DTT), 5 units of RNasin® Plus RNase inhibitor (Promega), 15 units of T7 RNAP (Ambion) and 0.5 µg of the linearized pTRI-β-actin-Mouse plasmid. Reactions were initiated by the addition of different combinations of NTPs at 100 µM each [e.g. * (ATP, GTP, CTP, UTP); −A (GTP, CTP, UTP); −G (ATP, CTP, UTP); and −U (ATP, GTP, CTP)], as well as with unbalanced mixtures of the four NTPs, including one that is added in a low concentration [e.g. −A^+^ (GTP, CTP and UTP at 100 µM each, plus 1 µM ATP); or −G^++^ (ATP, CTP and UTP at 100 µM each, plus 20 µM GTP)], always in presence of 0.2 µCi of [α-^32^P]CTP (Perkin Elmer). High-purity ribonucleotides were from GE Healthcare (cat. no. 27-2025-01). Reactions were quenched at appropriate times by adding the stop solution [10 mM ethylenediaminetetraacetic acid (EDTA) in 90% formamide, containing 3 mg/ml xylene cyanol FF and 3 mg/ml bromophenol blue]. The RNA products were resolved on denaturing polyacrylamide gel electrophoresis (20% polyacrylamide and 8 M urea). Then, gels were exposed to a phosphor screen and scanned with a BAS1500 phosphorimager instrument (Fuji).

### Single-nucleotide incorporation assays

The transcription (DNA-dependent RNA synthesis) fidelity of T7 RNAP was studied by single-nucleotide incorporation assays with correct and incorrect nucleotides at pH 7.9 in the presence of 6 mM MgCl_2_, and at pH 6.75 in the presence of 1.5 mM MgCl_2_. Assays were carried out with the VSR10 template-primer^56^, a hybrid containing double-stranded DNA and a short RNA oligonucleotide, but lacking the T7 RNAP promoter (Fig. 1). Template DNA, non-template DNA and primer RNA were mixed at a 1:1.5:1 ratio, heated at 95 °C for 20 min, and then progressively cooled at 75, 55, 45, 20 and 4 °C for 20 min each. The 10-nt primer RNA was previously labelled at its 5′ end with [γ-^32^P]ATP (Perkin Elmer) and T4 polynucleotide kinase (New England Biolabs) in 70 mM Tris-HCl buffer pH 7.6, containing 10 mM MgCl_2_ and 5 mM DTT. Before annealing, the labelled RNA primer was purified with a Micro bio-Spin^TM^ column (BioRad) loaded with Sephadex G-25 (GE Healthcare) to eliminate the excess of labelled ATP that could interfere with the transcription reaction.

Kinetic parameters for UTP and CTP incorporation were determined at 37 °C using a rapid quench-flow instrument (model QFM-400, Bio-Logic Science Instruments, Claix, France), upgraded with a mixer cross and a special mixer (Bio-Logic). Assays were carried out in 24 µl of 40 mM Tris-HCl buffer pH 7.9, containing 6 mM MgCl_2_ (or Bis-Tris buffer pH 6.75 with 1.5 mM MgCl_2_), 10 mM NaCl and 10 mM DTT, in the presence of increasing concentrations of NTP. The concentration of VSR10 was 240 nM and the recombinant T7 RNAP was added to a final concentration of 1 µM. Reactions were stopped with EDTA (0.3 M final concentration). Pre-steady-state kinetic data were fit to a burst equation: [P] = A × [1 − exp(−*k*_obs_ × *t*)] + *k*_ss_ × *t*, where [P] is the product concentration, A is the amplitude of the burst, *k*_obs_ is the apparent kinetic constant of formation of the phosphodiester bond and *k*_ss_ is the kinetic constant of the steady-state linear phase. The dependence of *k*_obs_ on the NTP concentration is described by the equation: *k*_obs_ = *k*_pol_ × [dNTP]/(*K*_d_ + [dNTP]), where *K*_d_ is the equilibrium constant and *k*_pol_ is the catalytic rate constant of the nucleotide incorporation reaction. Kinetic parameters were determined using curve-fitting tools provided by the SigmaPlot software (Systat Software Inc.).

The incorporation of non-complementary nucleotides GTP and ATP was performed manually with template-primer VSR10 (240 nM). Reactions were carried out in 20 µl of 40 mM Tris-HCl buffer pH 7.9 (or Bis-Tris buffer pH 6.75), containing 6 mM MgCl_2_ (or 1.5 mM MgCl_2_), 10 mM NaCl, 10 mM DTT, 2 mM spermidine and 24 units of RNasin® Plus RNase inhibitor. The T7 RNAP was supplied at 1 µM. Samples were preincubated at 37 °C for 10 min, and the reaction was then initiated by adding increasing concentrations of NTP. Reactions were stopped at different times (0–30 s) by mixing an aliquot of 4 µl with an equal volume of stop solution. Samples were incubated at 90 °C for 10 min before loading in polyacrylamide-urea gels. Results were analysed by phosphorimaging and nucleotide incorporation data were fitted to the Michaelis-Menten equation, as described above.

### Binding affinity of T7 RNAP for template-primer VSR10

The equilibrium dissociation constant (*K*_d_) for T7 RNAP binding to the template-primer VSR10 was determined after pre-incubating the enzyme with increasing concentrations of the 5′-^32^P-labelled VSR10 (60 nM-2.3 µM) at 37 °C for 10 min and then initiating the incorporation reaction by adding UTP to a final concentration of 5 µM. Reactions were carried out in 20 µl of 40 mM Tris-HCl buffer pH 7.9 containing 6 mM MgCl_2_ (or Bis-Tris buffer pH 6.75 containing 1.5 mM MgCl_2_), and 10 mM NaCl, 10 mM DTT, 2 mM spermidine and 24 units of RNasin® Plus RNase inhibitor. The T7 RNAP was supplied at 0.75–1 µM in reactions carried out at pH 7.9 with 6 mM MgCl_2_. At pH 6.75 and 1.5 mM MgCl_2_, the T7 RNAP concentration was 120–250 nM. Aliquots of 4 µl were taken at 10, 20, 30 and 40 s, quenched with stop solution and analysed by denaturing polyacrylamide gel electrophoresis and phosphorimaging, as described above. The burst amplitudes (RT bound to template-primer at time zero) were plotted as a function of the template-primer concentration, and the data were fitted to the following quadratic equation:

$$[{\rm{E}}\bullet {\rm{T}}/{\rm{P}}]=0.5\times [({K}_{d}+{{\rm{E}}}_{{\rm{T}}}+{\rm{T}}/{\rm{P}})-\sqrt{{({K}_{d}+{{\rm{E}}}_{{\rm{T}}}+{\rm{T}}/{\rm{P}})}^{2}-4\times {{\rm{E}}}_{{\rm{T}}}\times {\rm{T}}/{\rm{P}}}]\,$$

where E_T_ and T/P are the total active enzyme and template-primer concentration used in the assay, respectively, and *K*_d_ is the equilibrium dissociation constant for T7 RNAP binding to the template-primer.

### M13mp2-based forward mutation assays

The fidelity of RNA-dependent DNA synthesis of retroviral RTs was determined using M13mp2-based forward mutation assays (Supplementary Fig. S1)^57^. Briefly, the dsDNA genome of the M13mp2 bacteriophage containing the promoter for T7 RNAP upstream the *lac*Zα gene (T7-M13mp2) was digested with FspI (New England Biolabs). Five or six µg of linearized T7-M13mp2 were used as template for transcription by T7 RNAP (either the recombinant enzyme or the commercially available polymerase from Promega). Reactions were carried out in a total volume of 50 µl. Two different transcription conditions were used: (i) 40 mM Tris-HCl buffer (pH 7.9), containing 6 mM MgCl_2_ and 0.5 mM of each NTP; and (ii) 40 mM Bis-Tris or PIPES buffer (pH 6.75), containing 1.5 mM MgCl_2_ and 0.2 mM of each NTP. In both cases, reactions also contained 10 mM NaCl, 10 mM DTT, 2 mM spermidine and 50–60 units of RNasin® Plus RNase inhibitor. Samples were incubated at 37 °C for 2 hours to obtain a 313-nucleotide RNA product. The T7-M13mp2 dsDNA template was then digested with 5–6 units of RQ1 RNase-free DNase (Promega) at 37 °C during 15 min, and the RNA product was purified by extraction with phenol:chloroform:isoamyl alcohol (25:24:1, by volume), followed by ethanol precipitation in the presence of sodium acetate.

The purified RNA was used as template in reverse transcription reactions carried out with WT AMV, MLV, HIV-1_BH10_ and HIV-1_ESP49_ RTs, and mutant O_K65R and O_K65R/V75I RTs. The cDNA synthesis reactions were carried out at 37 °C for 2 hours in 50 μl of 25 mM Tris-HCl buffer pH 8.0, containing 100 mM KCl, 2 mM DTT, 4 mM MgCl_2_, 250 μM of each dNTP and 100 nM RT (active site concentration). Each reaction contained 2 pmol of RNA, previously heated to 65–70 °C for 5 min, and primed with a two-fold molar excess of the Rtr174/18 oligonucleotide (5′-CTGTTGGGAAGGGCGATC-3′). The reactions were stopped by adding EDTA at a final concentration of 15 mM and incubated at 80 °C for 5 min. Six to ten reverse transcription reactions were performed for each enzyme. The RNA template used in reverse transcription was digested at 37 °C for 1 hour, after adding 1 µl of RNase Cocktail^TM^ containing RNase A and RNase T1 (Ambion). The obtained cDNA products were then purified by phenol extraction as described above. The cDNA was then phosphorylated with T4 polynucleotide kinase and 1 mM of ATP at 37 °C for 1 hour. The reaction was stopped after incubation at 65 °C for 5 min.

Finally, the cDNA was hybridised with gapped DNA (M13mp2 dsDNA lacking the *lac*Zα gene in one of the two strands of DNA) in a solution containing 300 mM NaCl and 30 mM sodium citrate. Gapped DNA was obtained as previously described^13^. The cDNA was added in excess over gapped DNA and the mixture was heated to 70–75 °C for 5 min and slowly cooled at room temperature. The annealed products were analysed by electrophoresis in 0.8% agarose gels. These hybrid products were electroporated into *E. coli* MC1061 cells, and the transformed cells were plated onto M9 medium-containing plates with 5-bromo-4-chloro-3-indolyl-β-D-galactopyranoside (X-Gal) and isopropyl-β-D-galactopyranoside (IPTG) with *E. coli* CSH50 lawn cells. In *E. coli*, the phosphorylation of the cDNA facilitates its ligation to the gapped DNA while repairing the nick occurring between positions +191 and +192 of the *lacZα* gene. Mutant frequencies were calculated as the ratio of mutant (light blue or colourless) plaques to the total number of plaques screened. Mutant phenotypes were confirmed by nucleotide sequencing of the phage replicative-form DNA using primer 5′ -GCTTGCTGCAACTCTCTCAG-3′ (Macrogen Inc., Seoul, South Korea).

Specific error rates were calculated as previously described^57^, but taking into account that the primer used for reverse transcription hybridised at positions +174 to +191 of the *lacZα* nucleotide sequence. Errors were detected at 123 template positions for base substitutions and 178 for frameshifts (38 at runs, and 140 at non-runs). Despite the different target size for base substitutions and frameshifts, we determined overall error rates to facilitate the comparison of different retroviral RTs by considering the 200 template positions where any phenotypic change could be detected. It should be noted that silent mutations and mutations that do not affect the β-galactosidase activity may not be detected in these assays. It has been estimated that this limitation results in a 2- to 3-fold underestimation of the actual mutation rates^58^. Statistical analysis about the differences in the proportion of mutations at specific sites in the spectra of different RTs was determined by using a two-tailed Fisher’s exact test, using the GraphPad software.

## Electronic supplementary material

Supplementary information
