# Supplementary material for: Transcriptional inaccuracy threshold attenuates differences in RNA-dependent DNA synthesis fidelity between retroviral reverse transcriptases
Source: Sci Rep. 2018 Jan 12;8:627. doi: 10.1038/s41598-017-18974-8 (PMC5766491; doi:10.1038/s41598-017-18974-8)
Supplement: Supplementary file 1 — Supplementary information [file 41598_2017_18974_MOESM1_ESM.pdf]

Supplementary information

Transcriptional inaccuracy threshold attenuates differences in RNA-dependent DNA synthesis fidelity between retroviral reverse transcriptases

Alba Sebastián-Martín, Verónica Barrioluengo, and Luis Menéndez-Arias

Centro de Biología Molecular "Severo Ochoa" (Consejo Superior de Investigaciones Científicas & Universidad Autónoma de Madrid), c/Nicolás Cabrera, 1, Campus de Cantoblanco, 28049 Madrid, Spain

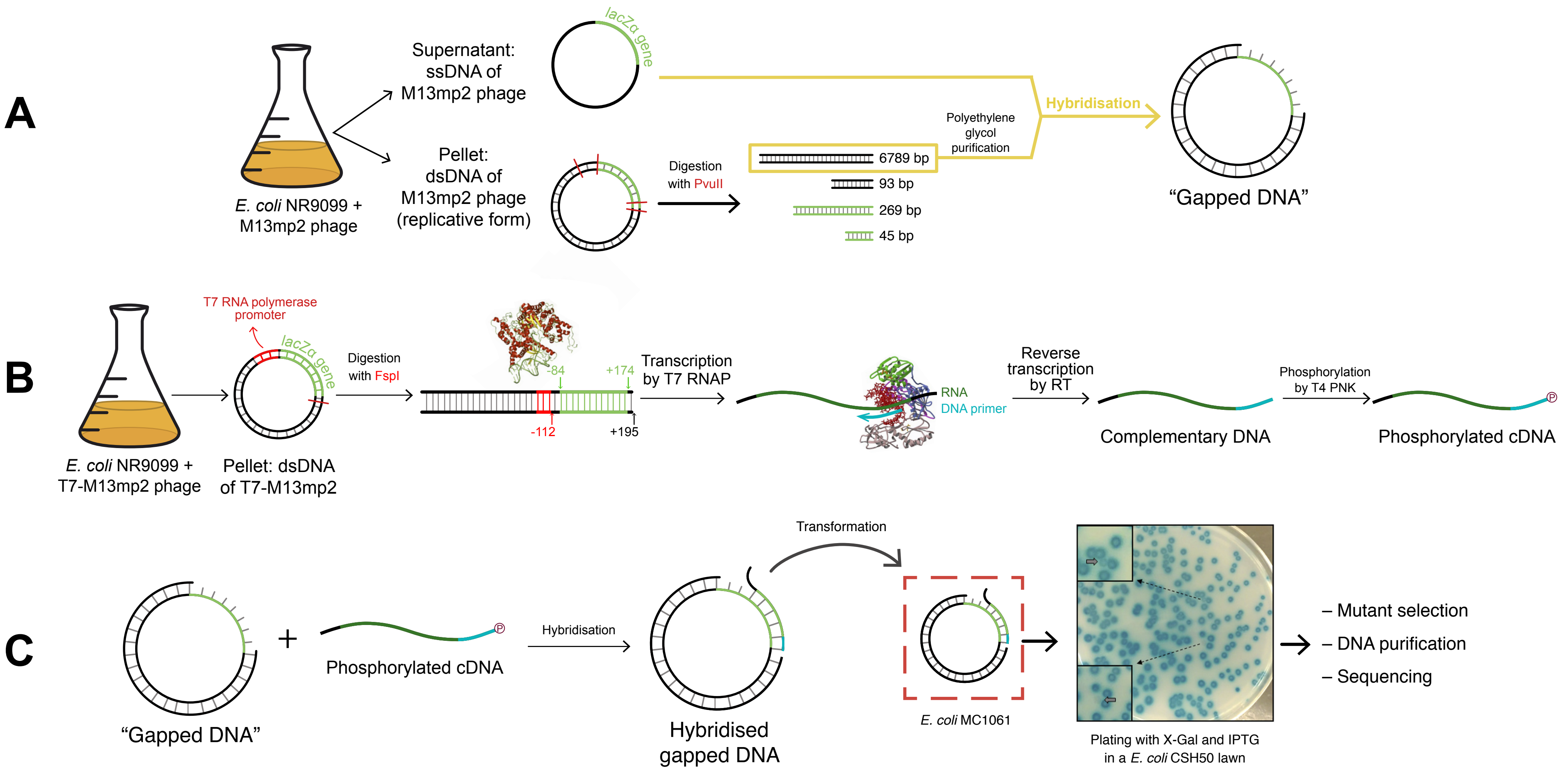

Supplementary Figure S1. Measurement of RNA-dependent DNA synthesis fidelity based on M13mp2 forward mutation assays. (A) Gapped DNA formation. Gapped DNA was obtained by hybridising the M13mp2 ssDNA with a 6789-bp dsDNA fragment. This fragment lacking the *lacZ*α sequence was obtained by polyethylene glycol purification after digestion of the M13mp2 dsDNA (or replicative form) with PvuII. (B) Synthesis of phosphorylated cDNA. Simultaneously, the dsDNA of a modified M13mp2 phage containing the promoter for T7 RNA polymerase upstream the *lacZ*α gene (T7-M13mp2) was purified. After its digestion with FspI, transcription with T7 RNA polymerase produces a 313-nt *lacZ*α transcript. This RNA was used as template for reverse transcription by different RTs, providing a cDNA product that was then phosphorylated. (C) Gapped DNA and phosphorylated cDNA were hybridised and the product was used to transform *Escherichia coli* MC1061 competent cells. The transformed cells were plated onto M9 medium-containing plates with 5-bromo-4-chloro-3-indolyl-β-D-galactopyranoside (X-Gal) and isopropyl-β-D-galactopyranoside (IPTG) with *E. coli* CSH50 cells. Errors made by RTs during reverse transcription result in a decrease in α-complementation and could be detected by the altered colour phenotype (light blue or colourless) of the mutant plaques.

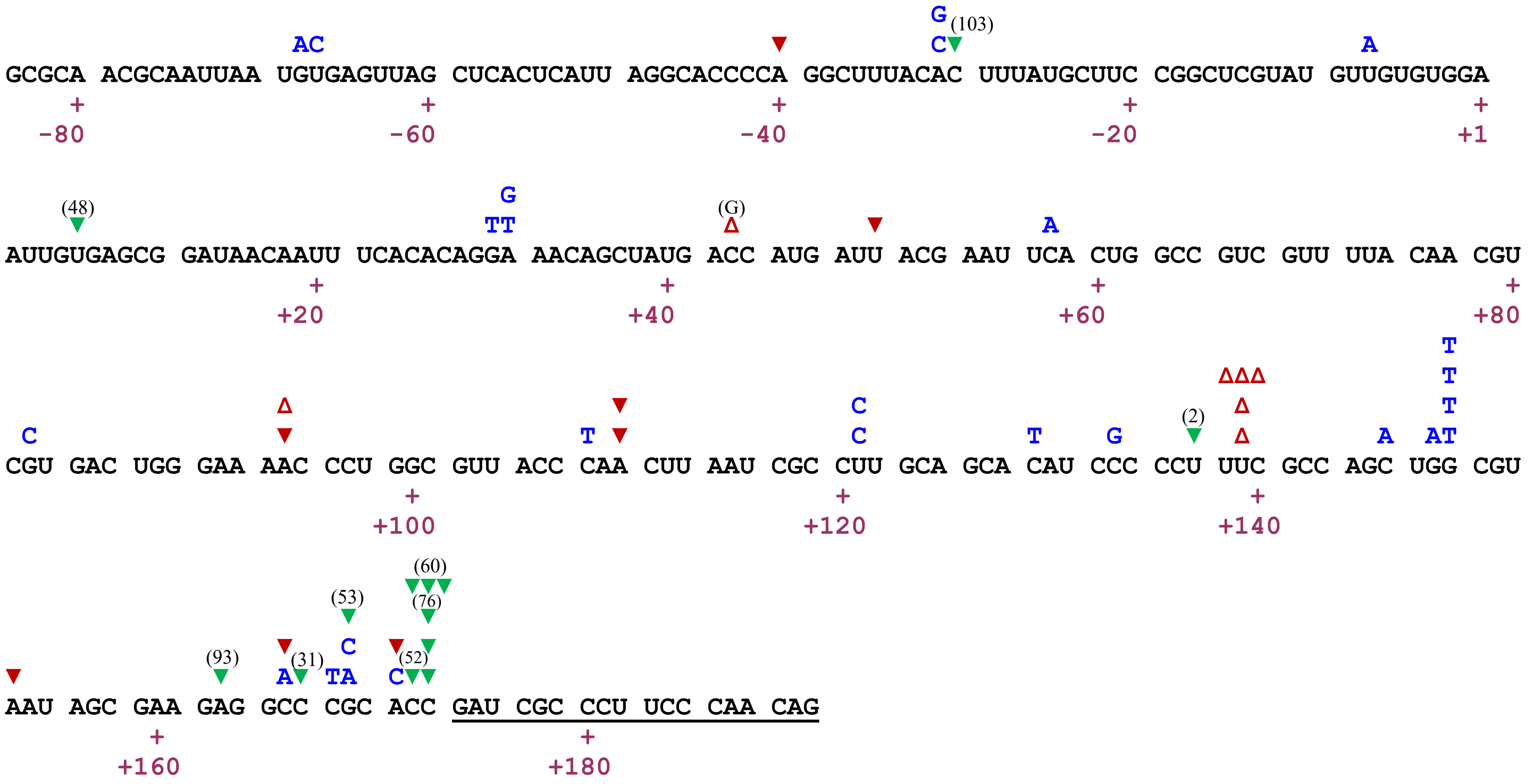

Supplementary Figure S2. Spectrum of mutations induced by HIV-1$_{BH10}$ RT in M13mp2 *lac*Z*α* forward mutation assays. The RNA used as template in the reverse transcription reaction was synthesized by the T7 RNA polymerase (Promega) at pH 7.9 (Tris-HCl buffer) and in the presence of 6 mM $MgCl_2$. Single-nucleotide substitutions are indicated by the letter corresponding to the new base (in blue) above the template sequence of the *lac*Zα target. Open upright red triangles represent insertions of one nucleotide (duplication of the base where the triangle is positioned, if not indicated). Inverted triangles indicate deletions of one (red) or more nucleotides (green). In the latter case, the triangle is positioned at the 3' end of the deletion and the number of deleted nucleotides is indicated above. Underlined bases represent the primer used for cDNA synthesis.

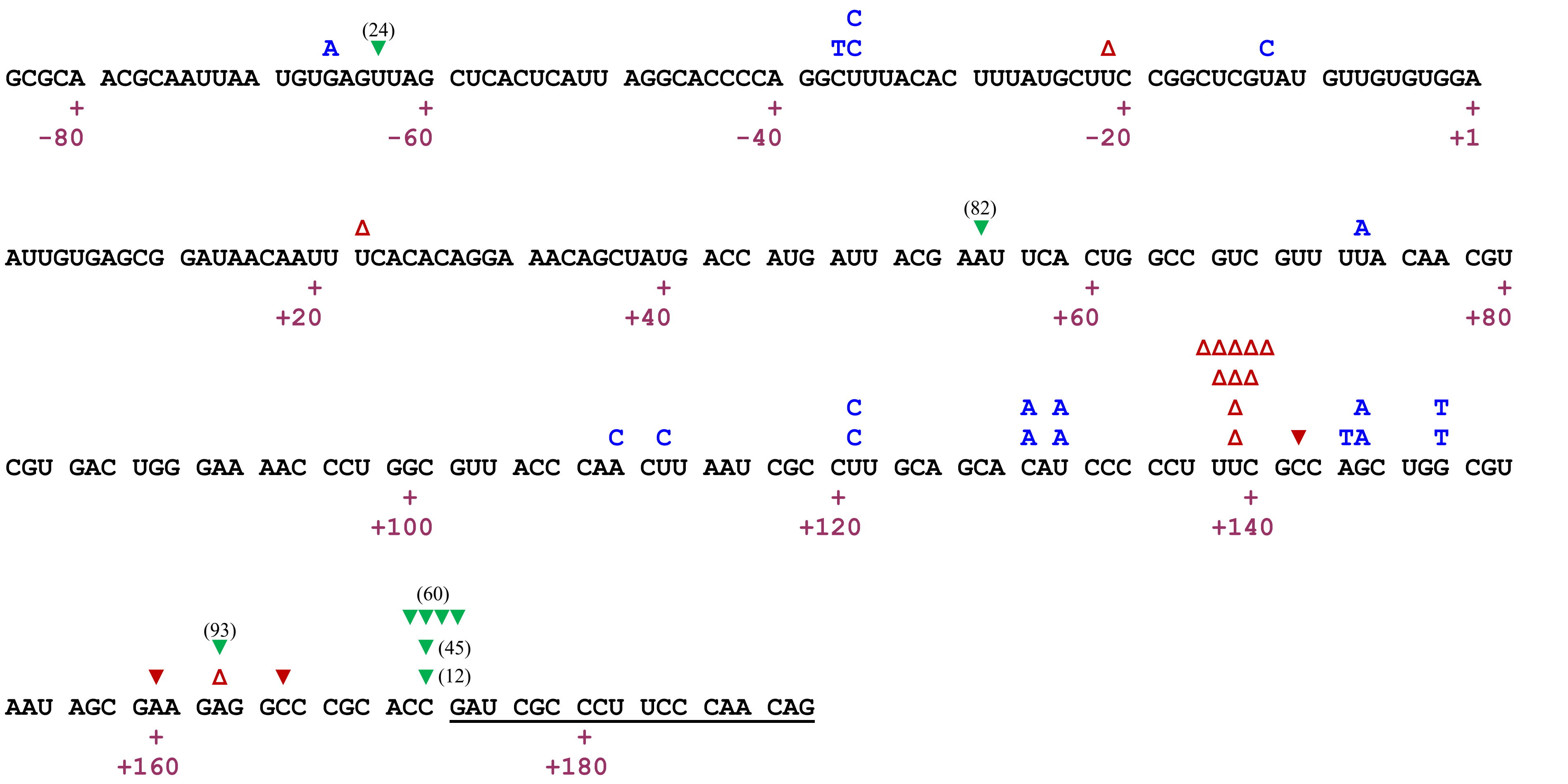

Supplementary Figure S3. Spectrum of mutations induced by HIV-1$_{ESP49}$ RT in M13mp2 *lacZα* forward mutation assays. The RNA used as template in the reverse transcription reaction was synthesized by the T7 RNA polymerase (Promega) at pH 7.9 (Tris-HCl buffer) and in the presence of 6 mM $MgCl_2$. Single-nucleotide substitutions are indicated by the letter corresponding to the new base (in blue) above the template sequence of the *lac*Zα target. Open upright red triangles represent insertions of one nucleotide (duplication of the base where the triangle is positioned). Inverted triangles indicate deletions of one (red) or more nucleotides (green). In the latter case, the triangle is positioned at the 3' end of the deletion and the number of deleted nucleotides is indicated above. Underlined bases represent the primer used for cDNA synthesis.

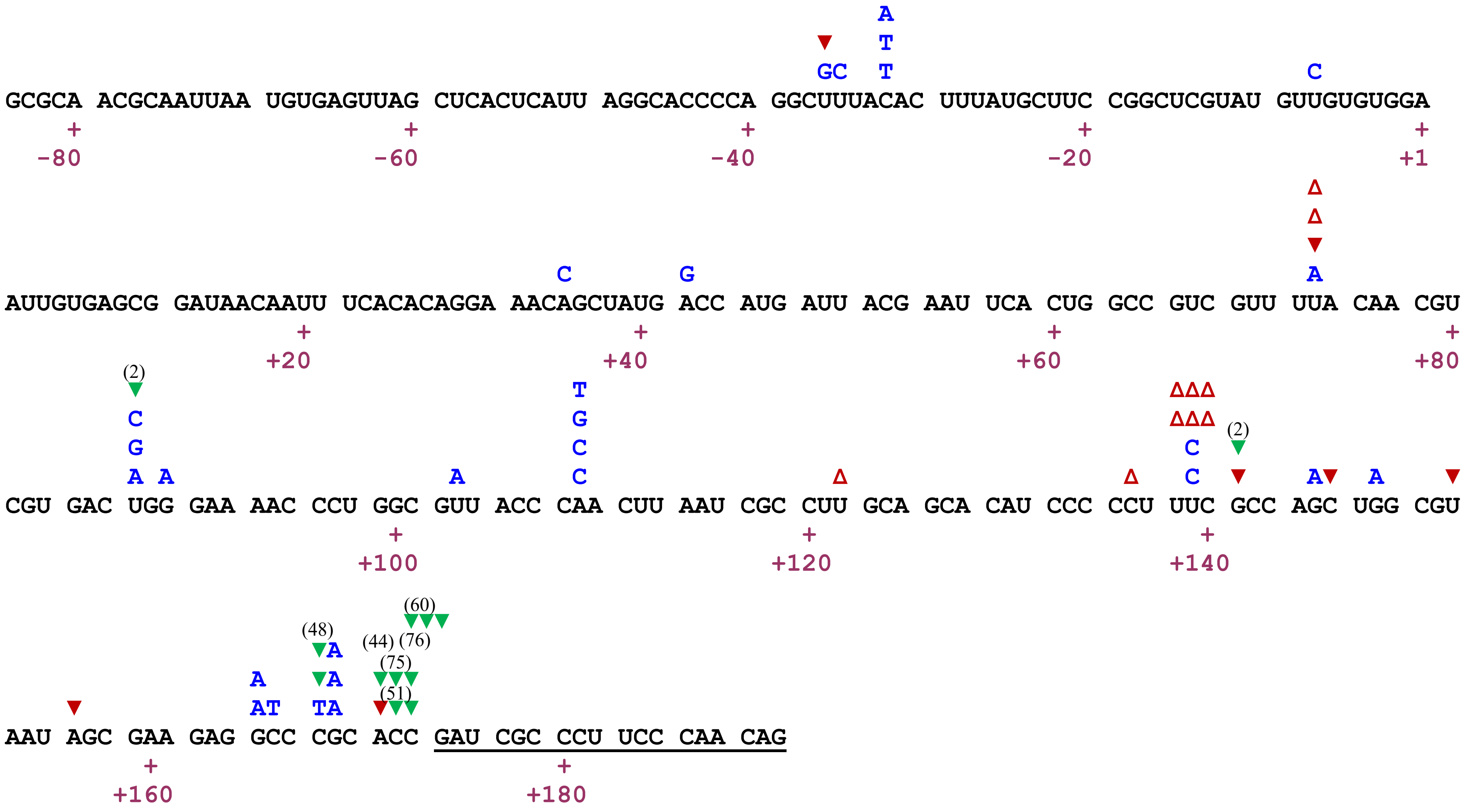

Supplementary Figure S4. Spectrum of mutations induced by O_K65R RT in M13mp2 *lacZα* forward mutation assays. The RNA used as template in the reverse transcription reaction was synthesized by the T7 RNA polymerase (Promega) at pH 7.9 (Tris-HCl buffer) and in the presence of 6 mM $MgCl_2$. Single-nucleotide substitutions are indicated by the letter corresponding to the new base (in blue) above the template sequence of the *lac*Zα target. Open upright red triangles represent insertions of one nucleotide (duplication of the base where the triangle is positioned). Inverted triangles indicate deletions of one (red) or more nucleotides (green). In the latter case, the triangle is positioned at the 3' end of the deletion and the number of deleted nucleotides is indicated above. Underlined bases represent the primer used for cDNA synthesis.

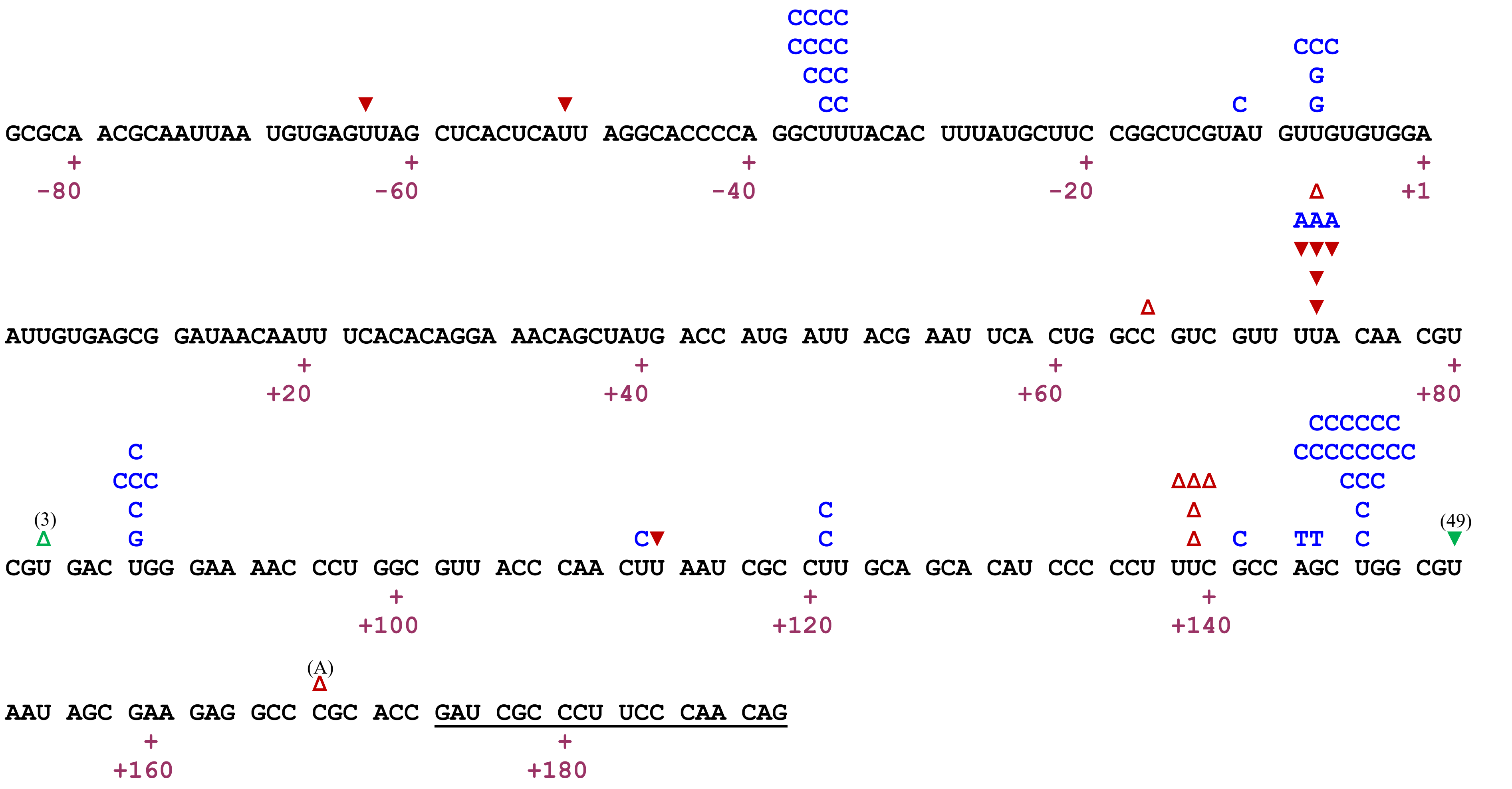

Supplementary Figure S5. Spectrum of mutations induced by O_K65R/V75I RT in M13mp2 *lac*Zα forward mutation assays. The RNA used as template in the reverse transcription reaction was synthesized by the T7 RNA polymerase (Promega) at pH 7.9 (Tris-HCl buffer) and in the presence of 6 mM $MgCl_2$. Single-nucleotide substitutions are indicated by the letter corresponding to the new base (in blue) above the template sequence of the *lac*Zα target. Open upright triangles represent insertions of one (red) or more nucleotides (green). In each case, the inserted base (if it is not a duplication of the template base) and the number of inserted bases are indicated in brackets. Inverted triangles indicate deletions of one (red) or more nucleotides (green). In the latter case, the triangle is positioned at the 3' end of the deletion and the number of deleted nucleotides is indicated above. Underlined bases represent the primer used for cDNA synthesis.

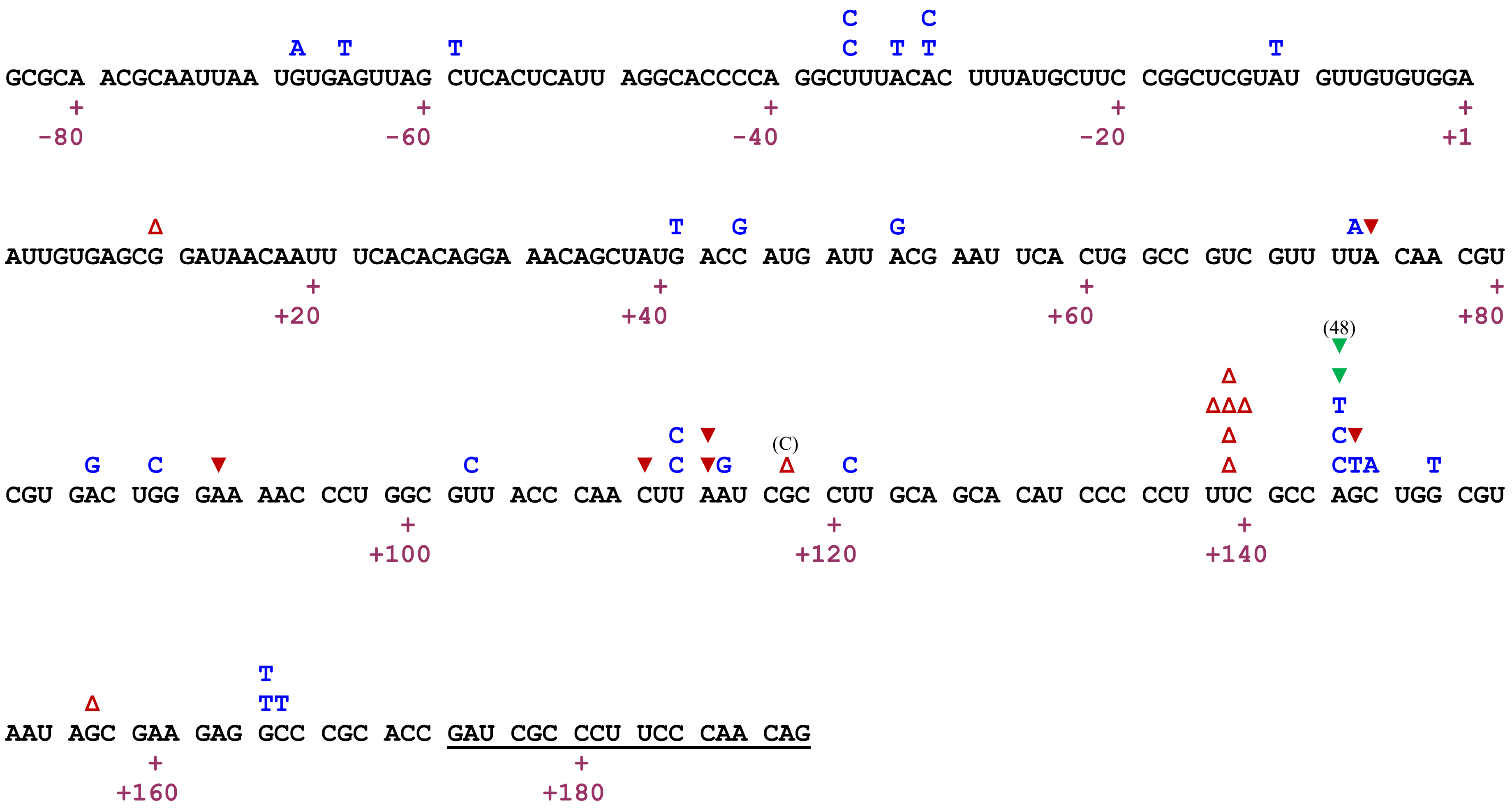

Supplementary Figure S6. Spectrum of mutations induced by MLV RT in M13mp2 *lac*Zα forward mutation assays. The RNA used as template in the reverse transcription reaction was synthesized by the T7 RNA polymerase (Promega) at pH 7.9 (Tris-HCl buffer) and in the presence of 6 mM $MgCl_2$. Single-nucleotide substitutions are indicated by the letter corresponding to the new base (in blue) above the template sequence of the *lac*Zα target. Open upright red triangles represent insertions of one nucleotide (duplication of the base where the triangle is positioned, if not indicated). Inverted triangles indicate deletions of one (red) or more nucleotides (green). In the latter case, the triangle is positioned at the 3’ end of the deletion and the number of deleted nucleotides is indicated above. Underlined bases represent the primer used for cDNA synthesis.

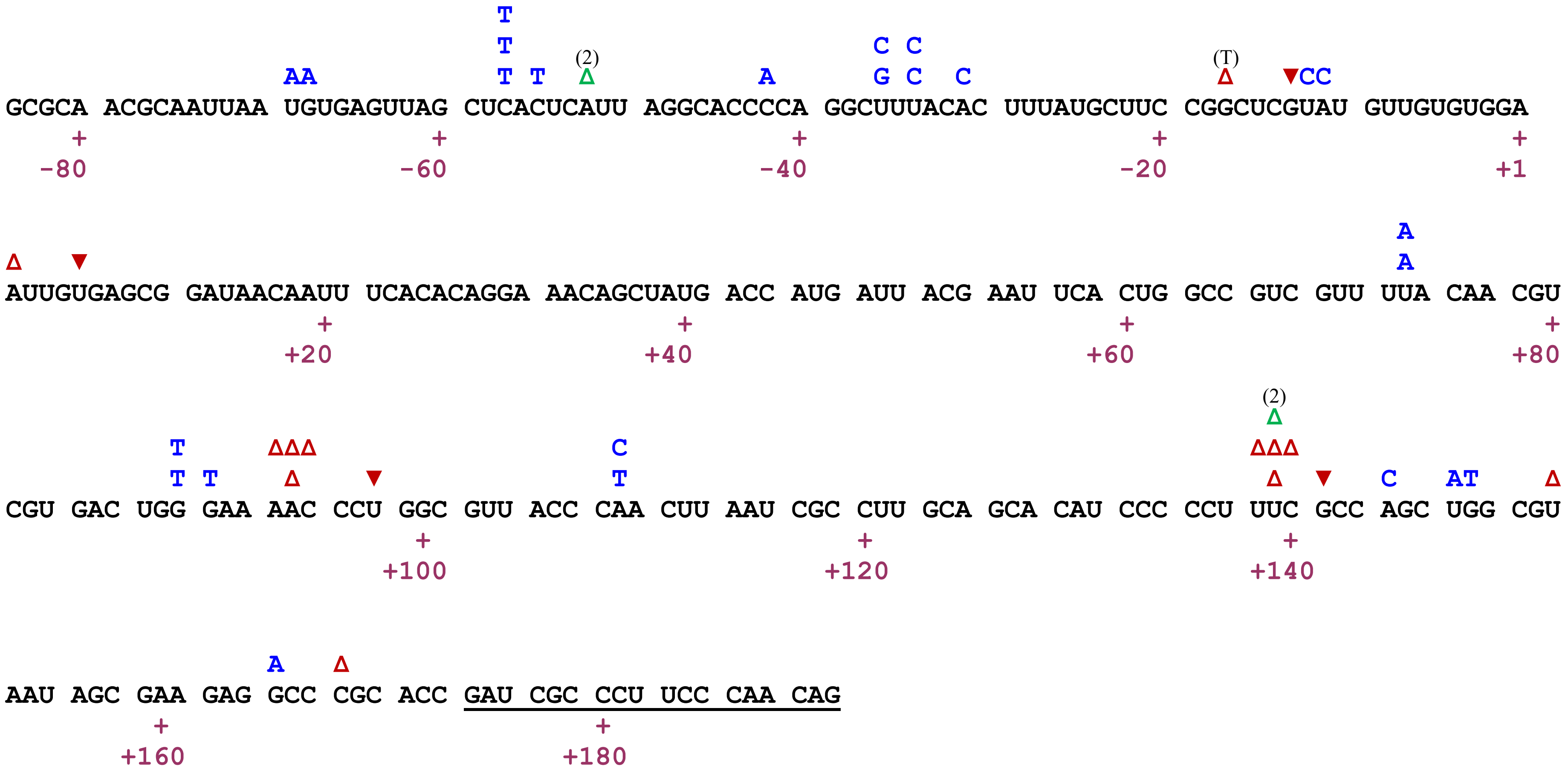

Supplementary Figure S7. Spectrum of mutations induced by AMV RT in M13mp2 *lacZα* forward mutation assays. The RNA used as template in the reverse transcription reaction was synthesized by the T7 RNA polymerase (Promega) at pH 7.9 (Tris-HCl buffer) and in the presence of 6 mM $MgCl_2$. Single-nucleotide substitutions are indicated by the letter corresponding to the new base (in blue) above the template sequence of the *lac*Zα target. Open upright triangles represent insertions of one (red) or more nucleotides (green). In each case, the inserted base (if it is not a duplication of the template base) and the number of inserted bases are indicated in brackets. Inverted triangles indicate deletions of one nucleotide (red). Underlined bases represent the primer used for cDNA synthesis.

Supplementary Table S1. Summary of mutational hotspots for WT and mutant RTs in M13mp2 *lacZα* forward mutation assays. The RNA used as template in the reverse transcription reaction was synthesized by the T7 RNAP (Promega) in a transcription buffer containing 40 mM Tris-HCl pH 7.9 and 6 mM $MgCl_2$. Listed hotspots correspond to sites where we found at least four mutations in any of the mutational spectra analysed.

	RTs						
	**HIV-1$_{BH10}$**	**AMV**	**MLV**	**HIV-1$_{ESP49}$**	**O_K65R**	**O_K65R/V75I**	
**Mutation type**	**No. of errors**	**No. of errors**	**No. of errors**	**No. of errors**	**No. of errors**	**No. of errors**	**Statistical analyses of hotspots [a]**
**All classes**	**54**	**43**	**46**	**44**	**58**	**71**	
**Base substitutions**							
**-36/-35**	**0**	**2**	**2**	**2**	**2**	**13**	***P*<0.002 and *P*<0.03 for the comparison of O_K65R/V75I RT *versus* HIV-1$_{BH10}$ and O_K65R RTs, respectively**
**-7**	**1**	**0**	**0**	**0**	**1**	**5**	**Differences not statistically significant**
**+87**	**0**	**0**	**0**	**0**	**3**	**6**	***P*<0.05 (O_K65R/V75I RT) *versus* HIV-1$_{BH10}$ RT**
**+109**	**0**	**2**	**0**	**0**	**4**	**0**	***P*<0.05 (O_K65R RT) *versus* O_K65R/V75I RT**
**+147**	**0**	**1**	**0**	**0**	**0**	**19**	**Strong hotspot in O_K65R/V75I RT (*P*<0.005 when compared with all other RTs)**
**+149**	**4**	**0**	**1**	**2**	**0**	**0**	***P*<0.05 (HIV-1$_{BH10}$ RT) *versus* O_K65R/V75I RT**
**Frameshifts**							
**+94**	**2**	**4**	**1**	**0**	**0**	**0**	***P*<0.04 (AMV RT) *versus* O_K65R and O_K65R/V75I RTs**
**+139**	**5**	**5**	**6**	**10**	**6**	**5**	**Differences not statistically significant**
**+173**	**6**	**0**	**0**	**6**	**5**	**0**	**Comparisons between HIV-1$_{BH10}$ and HIV-1$_{ESP49}$ RTs *versus* AMV, MLV and O_K65R/V75I RTs rendered *P* values in the range of 0.005 and 0.04. *P*<0.05 (O_K65R RT) *versus* O_K65R/V75I RT. Differences between HIV-1$_{BH10}$, HIV-1$_{ESP49}$ and O_K65R RTs were not significant, as well as differences between AMV, MLV and O_K65R/V75I RTs.**
**Both**							
**+73**	**0**	**2**	**1**	**1**	**4**	**9**	***P*<0.02 (O_K65R/V75I RT) *versus* HIV-1$_{BH10}$ RT**
**+144**	**0**	**1**	**5**	**1**	**0**	**1**	***P*<0.03 (MLV RT) *versus* HIV-1$_{BH10}$ and O_K65R RTs**

[a] Statistical analyses were carried out for the fifteen possible comparisons between the six RTs. The analysis was performed using a two-tailed Fisher's exact test.

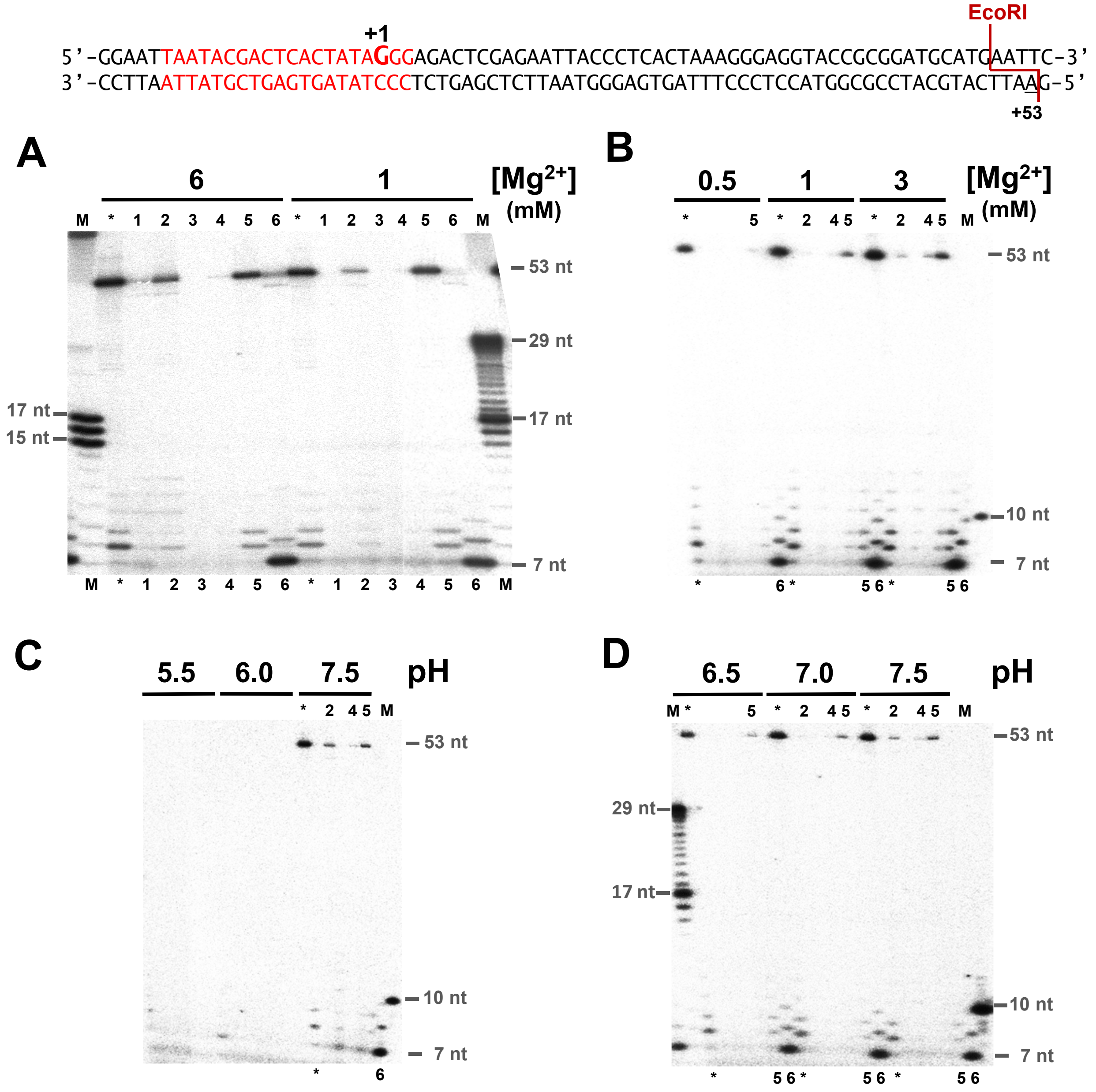

Panel	$[Mg^{2+}]$ (mM)	pH	Buffer	Time (min)
A	6	7.5	Tris-HCl	15
	1	7.5	Tris-HCl	15
B	0.5	6.75	PIPES	30
	1	6.75	PIPES	30
	3	6.75	PIPES	30
C	1	5.5	MES	30
	1	6.0	MES	30
	1	7.5	Tris-HCl	30
D	1	6.5	PIPES	30
	1	7.0	PIPES	30
	1	7.5	Tris-HCl	30

Lane	Symbol	rNTP composition[a]
*	*	U + A + G + C at 100 µM each
1	−A	U + G + C at 100 µM each
2	−A+	U + G + C at 100 µM each, plus 1 µM A [b]
3	−G	U + A + C at 100 µM each
4	−G+	U + A + C at 100 µM each, plus 10 µM G
5	−G++	U + A + C at 100 µM each, plus 20 µM G [c]
6	−U	A + G + C at 100 µM each

[a] Panel A (6 mM Mg^{2+}) contained 500 µM each NTP, instead of 100 µM.
[b] Panel A (6 mM Mg^{2+}) contained 2 µM ATP in lane 2.
[c] Panel A contained 50 µM GTP in lane 5.

Supplementary Figure S8. Promoter-dependent transcription by T7 RNAP in different reaction conditions. Part of the nucleotide sequence of plasmid pTRI-β-actin-Mouse (included in the MAXIscript® T7 Transcription Kit (Ambion)) including the T7 RNAP promoter (in red) and the EcoRI restriction site are shown above. After digestion with EcoRI, the plasmid was used as template for *in vitro* transcription by T7 RNAP in the presence of [α-^{32}P]CTP. The +1 base (G, in bold) is the first base incorporated into RNA during transcription. Then, the polymerase transcribes using the opposite strand (3'→5') as a template. Panels show transcriptions performed at different concentrations of Mg^{2+} (A and B) and at various pH (C and D). In control reactions (marked *), the four NTPs were supplied at 100 µM, except for reactions shown in panel A (6 mM Mg^{2+}) where their concentrations were increased to 500 µM each. Reactions carried out in the absence of A, G and U are shown in lanes 1, 3 and 6, respectively. Lanes 2, 4 and 5 show primer extensions carried out with limiting amounts of ATP and GTP (indicated in the tables below), and high concentrations of the three other NTPs. M stands for nucleic acid markers.

Supplementary Table S2. Nucleotide incorporation kinetics for GTP and ATP into VSR10 template-primer for recombinant T7 RNA polymerase.

NTP	pH 7.9 and 6 mM $MgCl_2$			pH 6.75 and 1.5 mM $MgCl_2$		
	k_{pol} (s^{-1})	K_d (μM)	k_{pol}/K_d (M^{-1} s^{-1})	k_{pol} (s^{-1})	K_d (μM)	k_{pol}/K_d (M^{-1} s^{-1})
GTP	(1.8 ± 0.7) x 10^{-2}	901.2 ± 334.9	19.77 ± 10.53	(4.3 ± 0.7) x 10^{-3}	652.5 ± 50.9	6.60 ± 1.16
ATP	(1.7 ± 0.2) x 10^{-2}	583.0 ± 296.6	29.15 ± 15.17	(4.1 ± 0.5) x 10^{-3}	367.8 ± 20.5	11.05 ± 1.53

Incorporation rates (k_{pol}) were determined by assuming the concentration of catalytically competent T7 RNAP in the assay (i.e. polymerase bound to template-primer at time zero) determined by using the dissociation equilibrium constants given in Figure 2. Data shown are the mean values ± standard deviations obtained from at least three experiments.

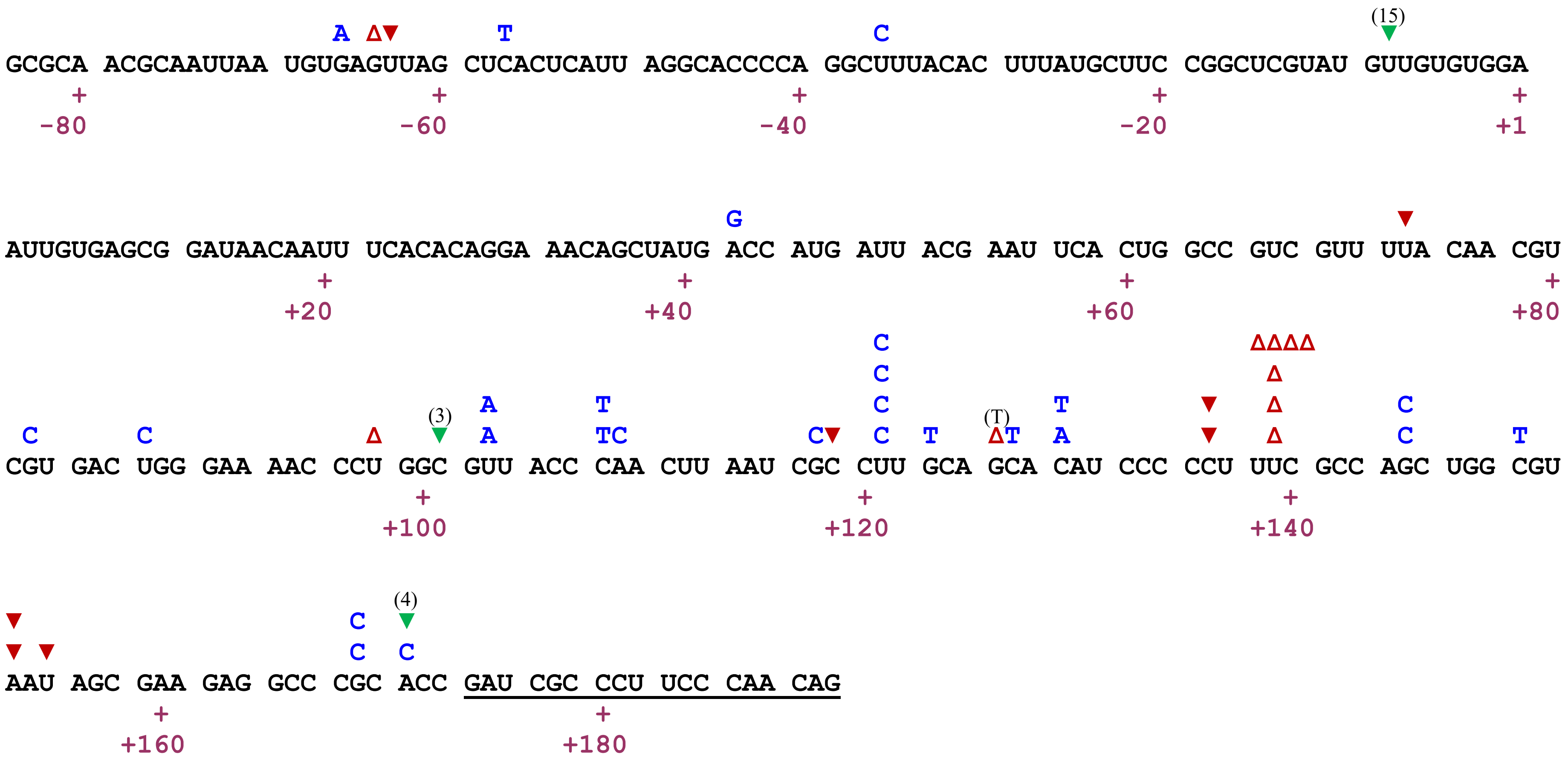

Supplementary Figure S9. Spectrum of mutations induced by HIV-1$_{BH10}$ RT in M13mp2 *lacZα* forward mutation assays. The RNA used as template in the reverse transcription reaction was synthesized by the recombinant T7 RNA polymerase at pH 7.9 (Tris-HCl buffer) and in the presence of 6 mM $MgCl_2$. Single-nucleotide substitutions are indicated by the letter corresponding to the new base (in blue) above the template sequence of the *lac*Zα target. Open upright red triangles represent insertions of one nucleotide (duplication of the base where the triangle is positioned, if not indicated). Inverted triangles indicate deletions of one (red) or more nucleotides (green). In the latter case, the triangle is positioned at the 3' end of the deletion and the number of deleted nucleotides is indicated above. Underlined bases represent the primer used for cDNA synthesis.

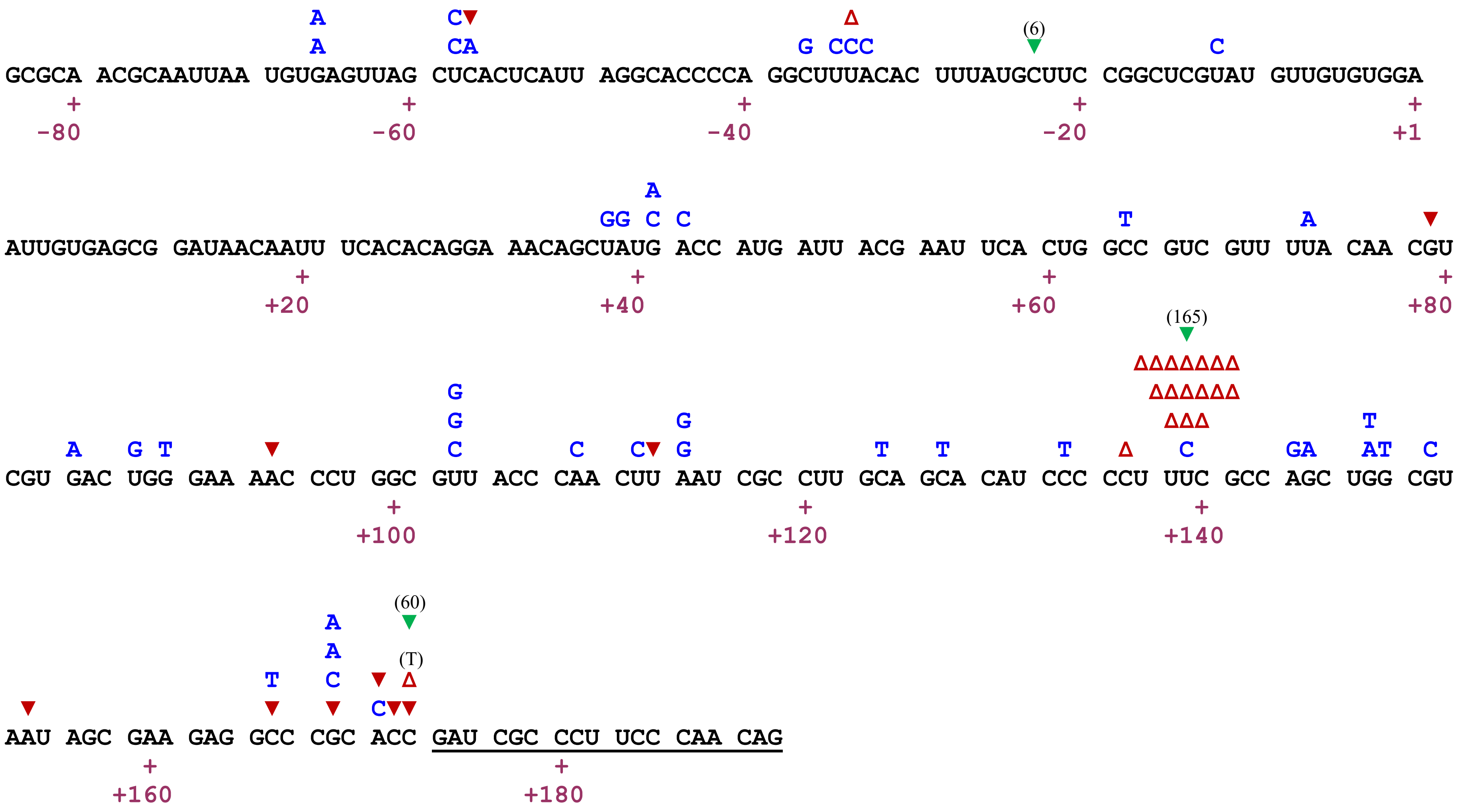

Supplementary Figure S10. Spectrum of mutations induced by HIV-1$_{BH10}$ RT in M13mp2 *lacZα* forward mutation assays. The RNA used as template in the reverse transcription reaction was synthesized by the recombinant T7 RNA polymerase at pH 6.75 (Bis-Tris buffer) and in the presence of 1.5 mM $MgCl_2$. Single-nucleotide substitutions are indicated by the letter corresponding to the new base (in blue) above the template sequence of the *lacZα* target. Open upright red triangles represent insertions of one nucleotide (duplication of the base where the triangle is positioned, if not indicated). Inverted triangles indicate deletions of one (red) or more nucleotides (green). In the latter case, the triangle is positioned at the 3' end of the deletion and the number of deleted nucleotides is indicated above. Underlined bases represent the primer used for cDNA synthesis.

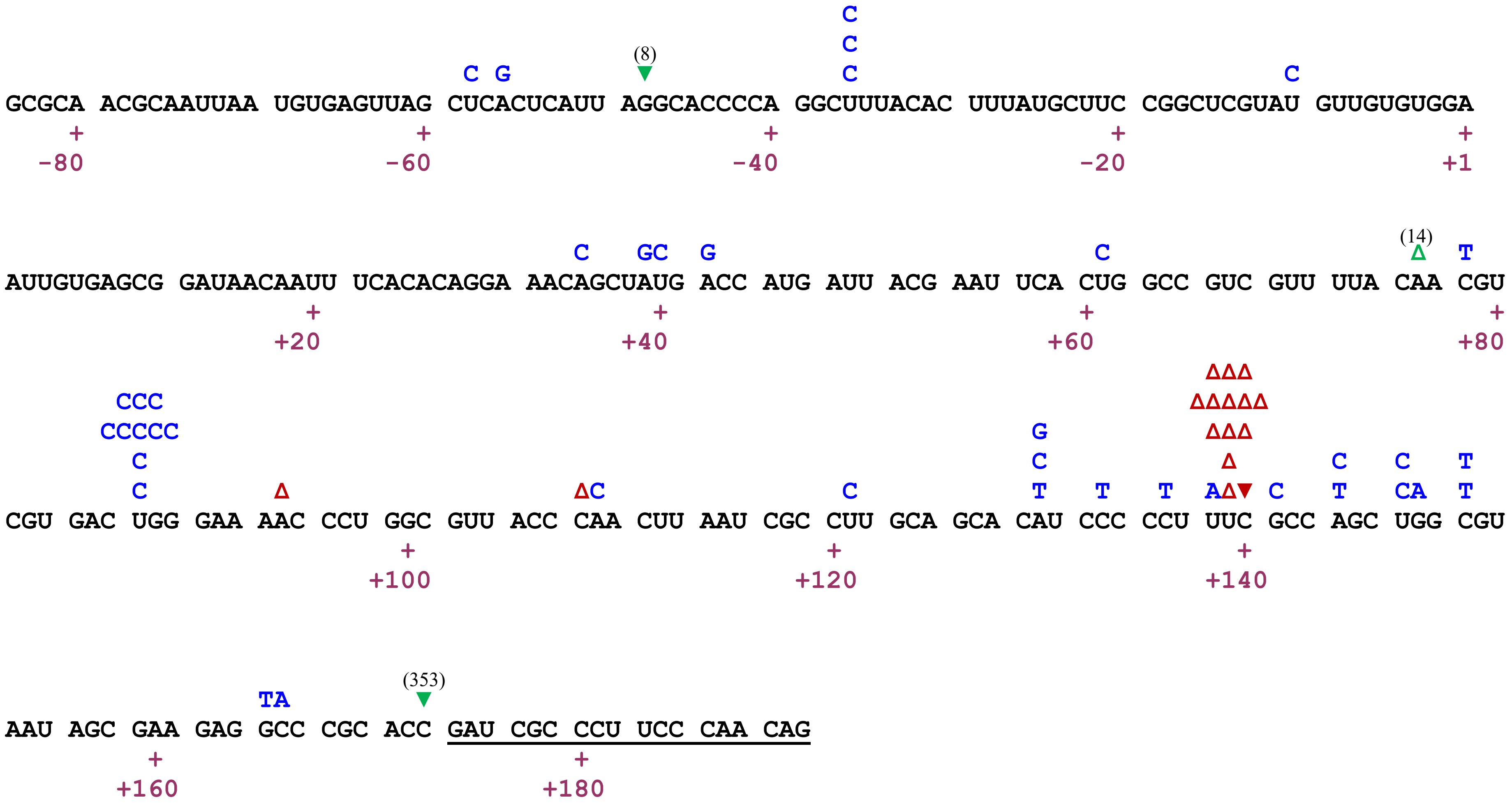

Supplementary Figure S11. Spectrum of mutations induced by O_K65R/V75I RT in M13mp2 *lacZα* forward mutation assays. The RNA used as template in the reverse transcription reaction was synthesized by the recombinant T7 RNA polymerase at pH 6.75 (Bis-Tris buffer) and in the presence of 1.5 mM $MgCl_2$. Single-nucleotide substitutions are indicated by the letter corresponding to the new base (in blue) above the template sequence of the *lacZα* target. Open upright red triangles represent insertions of one nucleotide (duplication of the base where the triangle is positioned), while open upright green triangles represent insertions of more than one nucleotide (the number of inserted bases is indicated in brackets). Inverted triangles indicate deletions of one (red) or more nucleotides (green). In the latter case, the triangle is positioned at the 3' end of the deletion and the number of deleted nucleotides is indicated above. Underlined bases represent the primer used during cDNA synthesis.

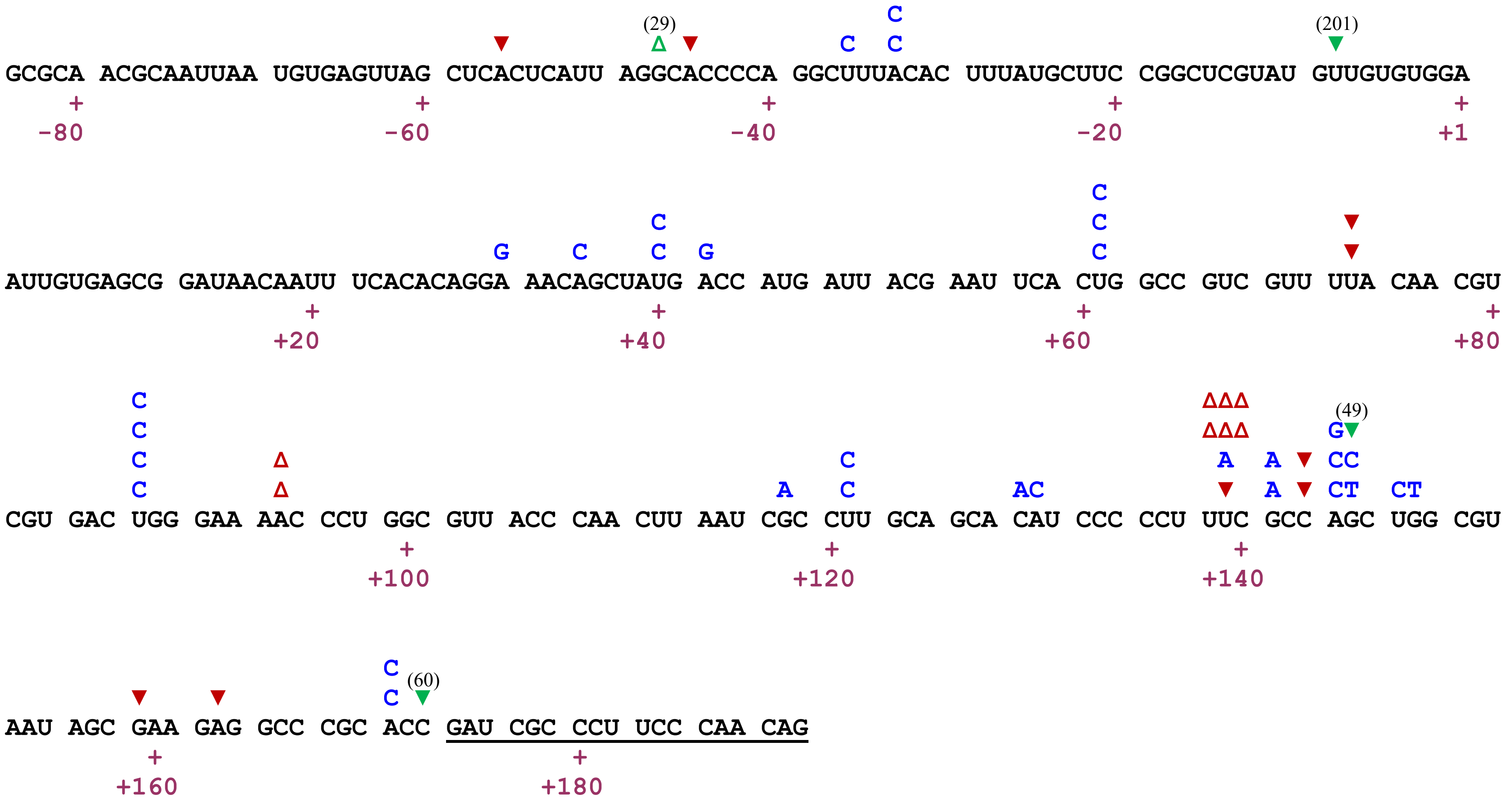

Supplementary Figure S12. Spectrum of mutations induced by O_K65R/V75I RT in M13mp2 *lacZα* forward mutation assays. The RNA used as template in the reverse transcription reaction was synthesized by the T7 RNA polymerase (Promega) at pH 6.75 (PIPES buffer) and in the presence of 1.5 mM $MgCl_2$. Single-nucleotide substitutions are indicated by the letter corresponding to the new base (in blue) above the template sequence of the *lacZα* target. Open upright red triangles represent insertions of one nucleotide (duplication of the base where the triangle is positioned), while open upright green triangles represent insertions of more than one nucleotide (the number of inserted bases is indicated in brackets). Inverted triangles indicate deletions of one (red) or more nucleotides (green). In the latter case, the triangle is positioned at the 3' end of the deletion and the number of deleted nucleotides is indicated above. Underlined bases represent the primer used for cDNA synthesis.
